# Centrosome/Cell Cycle Uncoupling and Elimination in the Endoreduplicating Intestinal Cells of *C. elegans*

**DOI:** 10.1371/journal.pone.0110958

**Published:** 2014-10-31

**Authors:** Yu Lu, Richard Roy

**Affiliations:** Department of Biology, The Developmental Biology Research Initiative, McGill University, Montreal, Quebec, Canada; University of North Carolina at Chapel Hill, United States of America

## Abstract

The centrosome cycle is most often coordinated with mitotic cell division through the activity of various essential cell cycle regulators, consequently ensuring that the centriole is duplicated once, and only once, per cell cycle. However, this coupling can be altered in specific developmental contexts; for example, multi-ciliated cells generate hundreds of centrioles without any S-phase requirement for their biogenesis, while *Drosophila* follicle cells eliminate their centrosomes as they begin to endoreduplicate. In order to better understand how the centrosome cycle and the cell cycle are coordinated in a developmental context we use the endoreduplicating intestinal cell lineage of *C. elegans* to address how novel variations of the cell cycle impact this important process. In *C. elegans*, the larval intestinal cells undergo one nuclear division without subsequent cytokinesis, followed by four endocycles that are characterized by successive rounds of S-phase. We monitored the levels of centriolar/centrosomal markers and found that centrosomes lose their pericentriolar material following the nuclear division that occurs during the L1 stage and is thereafter never re-gained. The centrioles then become refractory to S phase regulators that would normally promote duplication during the first endocycle, after which they are eliminated during the L2 stage. Furthermore, we show that SPD-2 plays a central role in the numeral regulation of centrioles as a potential target of CDK activity. On the other hand, the phosphorylation on SPD-2 by Polo-like kinase, the transcriptional regulation of genes that affect centriole biogenesis, and the ubiquitin/proteasome degradation pathway, contribute collectively to the final elimination of the centrioles during the L2 stage.

## Introduction

In many animal cells, the centrosome acts as the major microtubule organization center (MTOC) playing a key role in defining cell shape, cell division and overall microtubule geometry [1]. This MTOC function is of special importance in proliferating cells where the two centrosomes are responsible for accurately establishing the bipolar spindle. Therefore, centrosome function and its numeral integrity are essential for many organisms. Altering their numbers can lead to genomic instability and/or tumorigenesis [2]–[3].

The centrosome consists of a pair of barrel-shaped centrioles surrounded by pericentriolar material (PCM) [4]. During the centrosome cycle, the centrioles must disengage, duplicate, separate and undergo centrosome maturation, while each event takes place at an appropriate stage of the cell cycle under the control of stage-specific cell cycle kinases. A Polo-like kinase-1 (PLK-1) contributes to disengagement between the parental centrioles during M phase and consequently licenses centriole duplication in response to Cyclin-Dependent Kinase (CDK)-2 in the subsequent G1/S [5]–[6]. During G2/M, the parental centrioles further separate under CDK-1 and PLK-1 control [7]–[9]. The two centrosomes undergo maturation by recruiting PCM, consequently increasing the microtubule organizing capacity of the centrosome. This process is regulated by M phase kinases, such as PLK-1 and Aurora A kinases [10]–[11].

Centriole assembly is rate-limiting during duplication. In *C. elegans* it involves the sequential recruitment of many proteins that are conserved from invertebrates to humans [12]–[15]. Among these, a coiled-coil protein called SPD-2 plays a critical role in the process being the first of several proteins that localize to the mother centriole during centriole biogenesis. It is thereafter joined by ZYG-1, a protein kinase that is likely analogous to PLK-4 [16]–[19]. SAS-6, a probable ZYG-1 target [20]–[21] joins the complex thereafter with SAS-5 to assemble the “central tube” structure. Finally SAS-4 will be recruited to regulate microtubule attachment onto the central tube [12]–[13], [22]–[24].

Because various enzyme activities that drive cell division are also required for centriole duplication, the two processes are considered to be “coupled”. However, such coupling can be altered in various contexts. For example, in some respiratory epithelia hundreds of centriole-derived organelles that are critical for ciliogenesis called basal bodies are generated spontaneously without any requirement for DNA replication [25]–[26]. The converse is also true in the endocycling follicle cells of the *Drosophila melanogaster* egg chamber, wherein the centriole does not duplicate with each round of S phase and is eventually eliminated [27]–[28]. In each of these developmental contexts centriole duplication must be uncoupled from the cell cycle, yet how this uncoupling occurs remains poorly understood.

**Figure 1:**
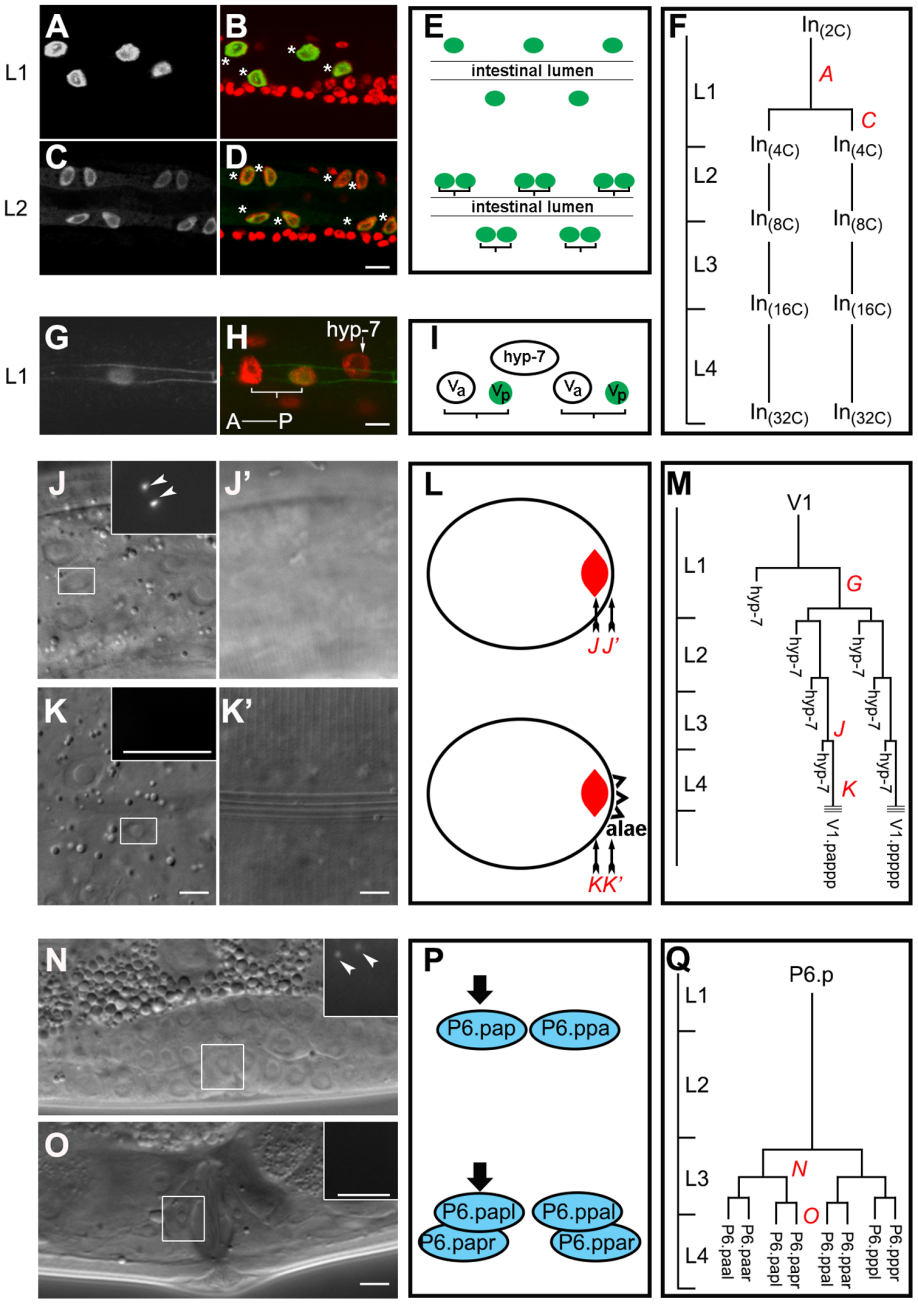
Centrioles are eliminated in many somatic cells of *C. elegans* following the completion of mitosis. (**A–D**) Larvae expressing intestine-specific *elt-2*::GFP were stained with DAPI (red) and anti-GFP (green) [44]. (A and C) show anti-GFP signal alone. Asterisks mark the intestinal nuclei. (**E**) A schematic diagram shows the relative position of intestinal nuclei before and after the nuclear division at the L1/L2 transition. Lineage brackets indicate two daughter cells from a common intestinal cell mother. Green ovals, intestinal nuclei. (**F**) A representative map of the postembryonic intestinal cell lineage: C refers to haploid DNA content in the nuclei. L1–L4 on the y axis indicate developmental timing showing the different larval stages; In, intestinal cells [29]–[30]. (**G** and **H**) Animals co-expressing the adherens junction marker *ajm-1*::GFP and seam cell marker *scm-1*::GFP [76] were stained with DAPI (red) and anti-GFP (green). (G) shows anti-GFP signal alone. Lineage brackets indicate two daughter cells from a common V cell mother. hyp7 and the arrow marks a hyp7 nucleus. A, anterior; P, posterior. (**I**) A schematic diagram summarizes the relative positions of V cells shown in (G) and (H). (**J–K′**) SPD-2::GFP was observed in the seam cells before, but not after the final cell division. White rectangles indicate the seam cells from the V1 lineage and the insets represent the magnified views of GFP signal in the corresponding white rectangles in J and K. J′ and K′ show the focal plane of the cuticle to indicate the presence or absence of adult alae (which indicates terminally differentiated seam cells in J and K), respectively. (**L**) A schematic diagram indicates the cross section view of a *C. elegans* body. Red spindles, V cell nuclei. The red italic letters and the black arrows together indicate the focal planes in the corresponding micrographs. (**M**) A map of the V1 lineage. The parallel lines indicate the alae/terminal differentiation. (**N** and **O**) SPD-2::GFP can be seen in the vulva cell lineage (P6.p) before (N) but not after (O) the completion of cell division. White rectangles highlight P6.p descendants and the insets represent the magnified views of GFP signal in the corresponding white rectangles. (**P**) A schematic diagram highlights later P6.p cell divisions a-anterior; p-posterior; l-left; r-right. Blue ovals depict nuclei of P6.p descendants. Black arrows point out the boxed nuclei in (N) or (O). (**Q**) A map of the P6.p cell lineage. The arrowheads indicate the SPD-2 foci. Scale bar, 5 µm. Red italicized letters in the lineage maps F, M, and Q show the precise time when the cells represented in the corresponding panels (non-italicized bold letters) were imaged.

In *C. elegans* both the intestine and the lateral hypodermal cells execute endocycles during larval development, giving rise to polyploid cells in the adult [29]. The intestinal nuclei undergo a single round of nuclear division in the absence of cytokinesis at the end of the first larval stage (L1) to become binucleate (Figure 1A–1E), followed by a single endocycle at the end of each larval stage [29] (Figure 1F). In the hypodermal V cell lineage, an anterior daughter cell is generated that undergoes endoreduplication and will eventually fuse with the hyp7 syncytium, while the posterior seam cell daughter will divide once during the L1 (Figure 1G–1I, 1M). After an equational division at the L1/L2 transition the V cell lineage repeats its L1 pattern of cell division in each subsequent larval stage, yielding one anterior endocycling cell that fuses with the hypodermis and its sister that will continue to execute a mitotic stem cell division [29] (Figure 1M).

Because the endocycling cells undergo reiterative rounds of DNA replication, it is unclear how the centrioles would respond to these successive rounds of S-phase-associated enzyme activity. We therefore used the postembryonic intestinal cell lineage as a model to determine the fate of centrioles in these endocycling cells and found that the centrioles lose their PCM following the nuclear division that occurs during the L1 stage and never regain it thereafter. Centriole duplication then becomes uncoupled from the first S-phase of the endocycles (endo-S), which precedes their elimination later during the L2 stage. We show that SPD-2, an important centriolar and pericentriolar component, may play a central role in the numeral regulation of centriole duplication, while transcriptional regulation of genes that affect centriole biogenesis, concomitant with the timely function of the ubiquitin/proteasome degradation pathway, contribute to the final elimination of the centrioles during the L2 stage.

## Results

### The centriole is eliminated in endocycling cells

During post-embryonic development in *C. elegans*, cells in both the hypodermal V cell and the intestinal E lineage execute endocycles to generate polyploid cells [29]–[30]. The successive cycles of DNA replication that are characteristic of the endocycle are driven by canonical S-phase regulators, many of which have previously been shown to trigger centriole duplication during the mitotic cell cycle [5], [31]–[32]. If centrosome duplication remains sensitive to the S-phase CDK activity the centrioles could potentially duplicate at each S-phase, resulting in an accumulation of centrioles in the polyploid adult cells. Alternatively, the centrioles might behave as they do in the *Drosophila* follicle cells, and become “uncoupled” from the endo-S-phase activities to be subsequently eliminated [28]. We therefore determined the centriole numbers/fate in the polyploid cells of *C. elegans* to distinguish between these possibilities.

**Figure 2:**
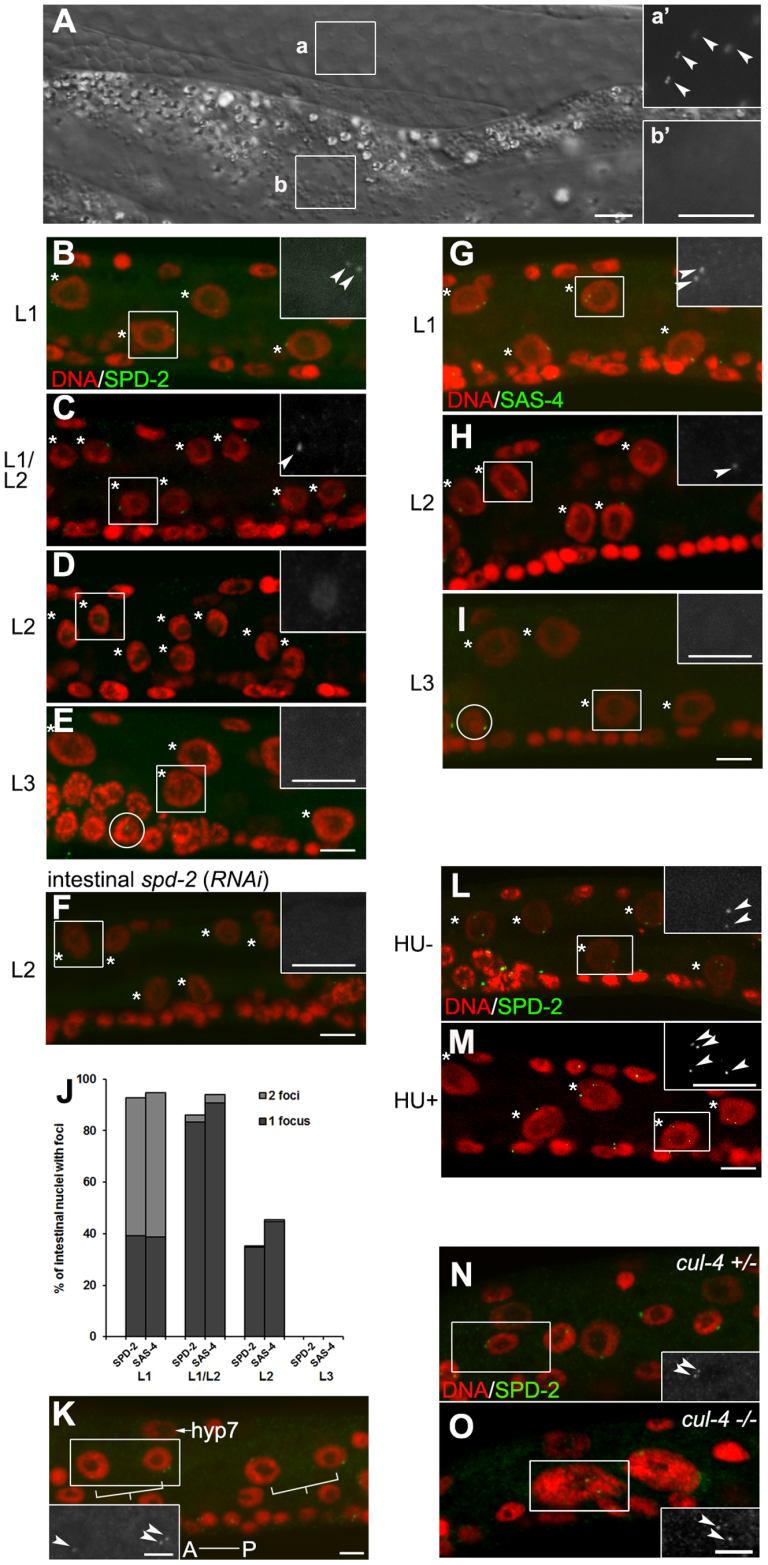
Centrioles no longer duplicate in endocycling cells prior to their elimination. (**A**) SPD-2::GFP signal can be seen throughout the germ line until oogenesis in (a), while in (b) it is undetectable in the intestinal cells in adult hermaphrodites. a′ and b′ are high magnification images of the GFP signal in the field identified by the two white rectangles a and b. (**B–F**) and (**G–I**) SPD-2 and SAS-4 foci are detectable up to the L1/L2 transition. The signal is no longer detectable by the L3 stage. The nuclear localized SPD-2 is absent following *spd-2*(*RNAi*) in (F). Animals were stained with DAPI (red) and anti-SPD-2 (green) in (B–F) or anti-SAS-4 (green) in (G–I). Arrowheads indicate centrioles, while asterisks indicate intestinal nuclei. The insets represent magnified SPD-2 or SAS-4 signal of the highlighted regions (white rectangles). The circles in (E) and (I) highlight the germ cells with either SPD-2 foci or SAS-4 foci. (**J**) Quantification of centriole numbers and described in (B–E) and (G–I) based on SPD-2 or SAS-4 detection. n≥56 for each stage. (**K**) Centriole duplication is uncoupled from the endocycles in the lateral hypodermal V cells in the late L1 stage. The centriole appears to be uncoupled from S-phase in the anterior endoreduplicating daughter cell but remains coupled to DNA replication in the mitotic posterior daughter. The square brackets indicate the daughter cells from a common mother V cell, while arrowheads indicate the centrioles. The inset is a magnified view of the region delineated by the white rectangle. A, anterior; P, posterior. hyp7 marks the hyp7 nucleus. (**L** and **M**) Supernumerary centrioles are detected in HU-treated L1 animals (M) but not in the control (L). The inset is a magnified SPD-2 signal of the region delineated by the white rectangle. Arrowheads indicate centrioles, while asterisks indicate intestinal nuclei. (**N** and **O**) Centriole duplication occurs once in response to S phase, but centrioles do not overduplicate and are not eliminated during un-quantized DNA re-replication. Heterozygous in (N) and homozygous *cul-4* (*gk434*) mutants in (O) were stained with DAPI (red) and SPD-2 (green) respectively. The insets show the SPD-2 signal in the regions outlined by the white rectangles. Arrowheads indicate centrioles. Scale bar, 5 µm.

We monitored the levels of two centriolar proteins in the intestinal cells throughout postembryonic development: SPD-2, which is associated both with the centriole and the PCM, and a highly conserved centriolar component called SAS-4 that is associated exclusively with centrioles [13], [33]. We first fused SPD-2 to GFP and found that it is most prominently expressed in the distal, mitotic region of the adult germ line (Figure 2A, a and a′), yet was notably absent from the adult intestinal cells (Figure 2A, b and b′), suggesting that SPD-2 was either not expressed in the adult intestinal lineage, and/or it was eliminated during development.

We therefore examined SPD-2 expression at each stage in the postembryonic intestine using the L1 nuclear division as a developmental landmark to discern between the L1 and L2 stage, since it represents the end of the mitotic cell cycle program. In wild type larvae, throughout the L1 stage and until ∼3 hours after the nuclear division, SPD-2 was present at the centrioles in the majority of the intestinal cells (Figure 2B, 2C, 2J). However, SPD-2 became undetectable during progression through the L2 stage: only half of the intestinal nuclei possessed SPD-2 foci 6–8 hours after the nuclear division (Figure 2J). This progressive loss of SPD-2 precedes its complete elimination by the L3 stage (Figure 2E, 2J).

In parallel, like SPD-2, SAS-4 was associated with intestinal nuclei throughout the L1 and during the L1/L2 transition before becoming undetectable by the end of the L2 (Figure 2G–2I, 2J), suggesting that the loss of SPD-2 signal during the L2 faithfully represents the fate of the centriole. Taken together, our observations reveal that the centrioles are still present until the completion of the nuclear division in the L1, but are progressively eliminated during the L2 stage in the intestinal cells.

### Centriole duplication becomes uncoupled from S phase in endocycling cells

The intestinal nuclear division is therefore a temporal landmark that not only marks the end of the mitotic cell division cycle in this lineage but also the onset of centriole elimination. Prior to the intestinal nuclear division, two SPD-2-positive foci are detectable in more than half of the intestinal nuclei 10–12 h into the L1 stage (Figure 2B, 2J), a period that corresponds to the final S-phase before the onset of the endocycle program [29]. The same intestinal nuclei also harbor two SAS-4-positive foci likely representing two pairs of centrioles that arose from a single round of duplication (Figure 2G, 2J). In order to confirm if the two SPD-2 foci result from centriole duplication during the final mitotic S-phase, we labeled cells with propidium iodide (PI) to quantify the DNA content in the intestinal nuclei, providing us with a pre-/post-S-phase reference point [34]. From our analysis we noted that single SPD-2 foci, most likely representing a pair of centrioles, were predominant in 2C intestinal cells (Figure S1A; average intestinal cell C value  = 1.98); whereas the majority of 4C intestinal cells contain SPD-2 doublets corresponding to two pairs of centrioles (Figure S1B; average intestinal cell C value  = 4.02), suggesting that centriole duplication is appropriately coupled during the final mitotic S-phase in the L1 intestinal cells.

After the nuclear division, only a single SPD-2 focus (1 centriole pair) is detectable adjacent to the majority of the 4C nuclei that are generated after the first endo-S phase (Figure 2C, 2J, S1C; average intestinal cell C value  = 3.98). Moreover, the numbers of SAS-4 foci are similar to the numbers of SPD-2 foci observed following the intestinal nuclear division (Figure 2H, 2J), suggesting that the centrioles do not duplicate after the division despite the initiation of initial S-phase associated with the first endocycle. Although we never see two SPD-2/SAS-4 foci after the nuclear division, we cannot formally rule out that the centrioles indeed duplicate but never separate, making it impossible to resolve them by standard confocal microscopy. The intestinal nuclear division is therefore a critical landmark that is associated with a potential change in the ability of the centriole to duplicate in response to the surrounding S-phase CDK activity. This apparent refractoriness may be a general feature of endoreduplicating cells, since a similar SPD-2 singlet was present in the anterior endoreduplicating daughter cells of the V cell lineage, whereas a doublet was observed in the posterior seam cell nuclei that do not undergo endocycles, but rather execute mitotic cell divisions (Figure 2K).

We also noticed that the majority of the SPD-2 signal transiently diffuses throughout the nuclei beginning at the L2, before the signal becomes undetectable later during the L2 stage (Figure 2D). This nuclear signal disappears following *spd-2*(*RNAi*), demonstrating that this signal is indeed SPD-2-specific (Figure 2F). Similarly, in the germ line, where centrioles are eliminated at the onset of oogenesis [35]–[36], SPD-2 also becomes diffuse prior to the loss of centriolar markers (Figure S2). This transient change in SPD-2 localization appears to precede the elimination of the centrioles and may reflect specific modifications of SPD-2 that determine centriole stability in both the intestine and the germ line.

### The intestinal nuclear division is followed by a failure to recruit PCM to the centriole

**Figure 3:**
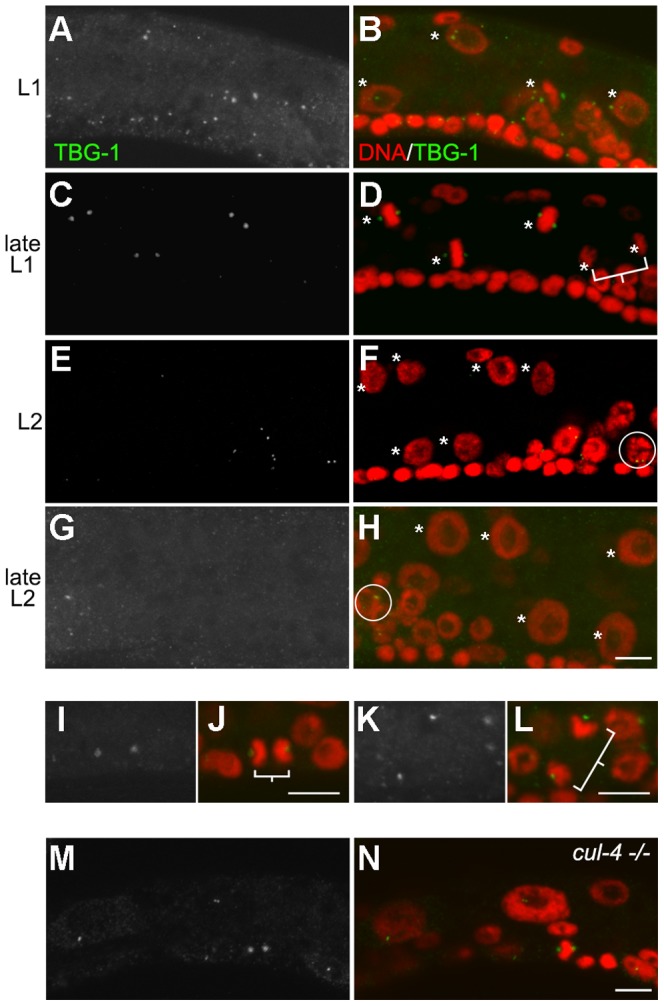
Centrioles lose their capacity to recruit γ-tubulin following the intestinal nuclear division that precedes the onset of endoreduplication. (**A–H**) Wild type larvae were stained for γ-tubulin (TBG-1) before, during, and after the intestinal nuclear division. (A), (C), (E) and (G) represent the TBG-1 only; (B), (D), (F), and (H) TBG-1 with DAPI. The asterisks indicate the intestinal nuclei and the square bracket highlights a pair of sister intestinal nuclei. The circles in (F) and (H) highlight germ cells with γ-tubulin foci. (**I–L**) High resolution micrograph of cells following division during the L1 stage. (I and K) show TBG-1 signal only; (J and L) TBG-1 with DAPI. (I and J), dividing P cell in the ventral hypodermis; (K and L), germ line precursor cell. Square brackets highlight sister cells. (**M** and **N**) Similar staining was performed in *cul-4* (*gk434*) homozygous animals. All cells were stained with DAPI (red) and anti-γ-tubulin (green). Scale bar, 5 µm.

One of the major functions of the centrosome is to organize the mitotic spindle during mitosis through recruitment of the γ-tubulin complex and other components of the PCM. The changes in centriole duplication and stability during the mitosis-endocycle transition led us to examine if any functional modification of the centrioles might also occur as a consequence of this process. Previous studies indicated that γ-tubulin recruitment is subject to cell cycle-dependent regulation in mitotic cells [37]. During centrosome maturation γ-tubulin accumulates around the centrioles, resulting in substantially enlarged γ-tubulin foci at metaphase. The intensity of γ-tubulin gradually returns to baseline levels at the onset of the next interphase [37]. We observed similar baseline levels of γ-tubulin expression during the L1 interphase (Figure 3A, 3B). The intensity of the γ-tubulin foci increases substantially when the metaphase chromosomes become discernable (Figure 3C, 3D; n = 15). During anaphase, most of the centrosomal γ-tubulin rapidly disperses prior to the onset of the following S-phase (Figure 3C, 3D). The anaphase dispersal of γ-tubulin is not due to the disappearance of the centriole (Figure S3A, a-a” and b-b”), since centriole elimination only begins later during the L2 stage (Figure 2J). Moreover, the γ-tubulin levels around the centrioles never recover thereafter (Figure 3E–3H). This change likely compromises the ability of the centrioles to function as a MTOC in intestinal cells following this stage (Figure S3B). This anaphase dispersal of γ-tubulin does not occur in the mitotically proliferating cells in the ventral hypodermis (Figure 3I, 3J), or in the germ cells that are simultaneously undergoing mitotic divisions (Figure 3K, 3L). Not surprisingly, centrioles still act as an MTOC in these mitotic cells (Figure S3C). Overall, our observations indicate that just subsequent to the intestinal cell nuclear division that occurs at late L1 stage, the γ-tubulin that is associated with the centriole disperses, thereafter compromising the ability of the centriole to act as a MTOC.

### Centriole response during unscheduled DNA synthesis

We noted that centrioles do not duplicate with each round of DNA replication in endocycling cells but are essentially refractory to the endo-S and are subsequently eliminated during the L2 stage. To determine whether the uncoupling of centriole duplication from S-phase activity and subsequent elimination is unique to endocycling cells, or alternatively a general response to reiterative or prolonged phases of DNA replication, we examined centriole behaviour during contexts of extended or misregulated S phase. First we used hydroxyurea (HU) to arrest DNA replication in cells during the L1 stage prior to the final mitotic S phase and then we tested the effects of HU just prior to the onset of the endocycle program [38]–[39]. Similar to what is observed in HU-treated vertebrate cells or in the blastomeres of the *C. elegans* embryo, a small population (∼15%) of intestinal cells that were HU-arrested before the nuclear division did indeed possess supernumerary centrioles (Figure 2L, 2M; Table 1), [38]–[40], while the centriole numbers indicate that only a single extra duplication event per centriole took place during this period (Figure 2L, 2M). Furthermore, the number of HU-blocked cells that possess supernumerary centrioles increased with time (data not shown) suggesting that a critical constraint to centriole duplication may be progressively relieved during prolonged S phase.

**Table 1:** % of supernumerary centrioles in HU treated animals.

Treated Stage	HU-	HU+
L1 (mitotic S arrest)	0	15.6±3.8*
L1/2 (endo-S arrest)	2.2±1.9	2.2±1.9**

The frequency of supernumerary centrioles is quantified by the number of intestinal nuclei with more than two SPD-2 foci during the L1 arrest or more than one SPD-2 focus during the L2 arrest.

*6-hour HU treatment on synchronized L1 animals.

**6-hour HU treatment on post L1 nuclear division larvae.

In contrast, when the same treatment was performed for a comparable duration after the nuclear division and prior to the onset of the endocycles, no detectable centriole amplification was observed compared to the control intestinal cells (Table 1). In all cases however, whether DNA replication was blocked before or after the nuclear division, following the release from HU treatment the centrioles were eliminated during the L2 stage.

Since the majority of the centrioles in the intestinal cells did not over-duplicate during a prolonged S phase we were curious whether the centrioles in other cell types might react in a similar way when the cycles of DNA replication and mitosis are disrupted. A mutation in *cul-4* stabilizes a positive regulator of a DNA replication licensing factor that causes the hypodermal cells of *cul-4* mutants to undergo un-quantized DNA replication yielding nuclei that contain >100 C DNA content (Figure 2O) [41]. We were therefore curious whether the centrioles would duplicate under conditions of re-replication in these hypodermal cells or whether the cells would respond to the re-replication by eliminating the centrioles. We monitored centriole numbers in the hypodermal seam cells of wild type and *cul-4* mutant larvae and found that like wild type animals the centrioles duplicate normally once at the first S-phase (Figure 2N), but are neither over-duplicated nor eliminated thereafter during the numerous phases of un-quantized re-replication (Figure 2O). Furthermore, the γ-tubulin levels in the *cul-4* hypodermal cells remain restricted to centriolar foci: they no longer fluctuate and are maintained at basal levels typical of interphase cells (Figure 3M, 3N). These observations suggest that the centrioles in *cul-4* cells undergo duplication normally in response to the first S phase, but do not duplicate in response to the signals associated with the inappropriate re-replication events that occur thereafter, nor do the centrioles mature to accumulate γ-tubulin. Because these hypodermal cells do not transit through mitosis after their initial S phase, they probably do not become properly licensed for subsequent centriole duplication [6], [42]. However, despite their inability to duplicate and or recruit PCM, these centrioles are never eliminated in the *cul-4* mutant hypodermal cells.

The elimination process we observe in the endocycling cells (both the anterior hypdodermal daughters and the intestinal cells) must therefore be dependent on the developmental fate of these cells and independent of the S phase program *per se* since the *cul-4* seam cell centrioles persist into later stages, while the centrioles in the intestinal cells are gradually eliminated following the onset of the endocycle program to become undetectable by the L3 stage, even in situations where the cells are blocked in S phase.

**Figure 4:**
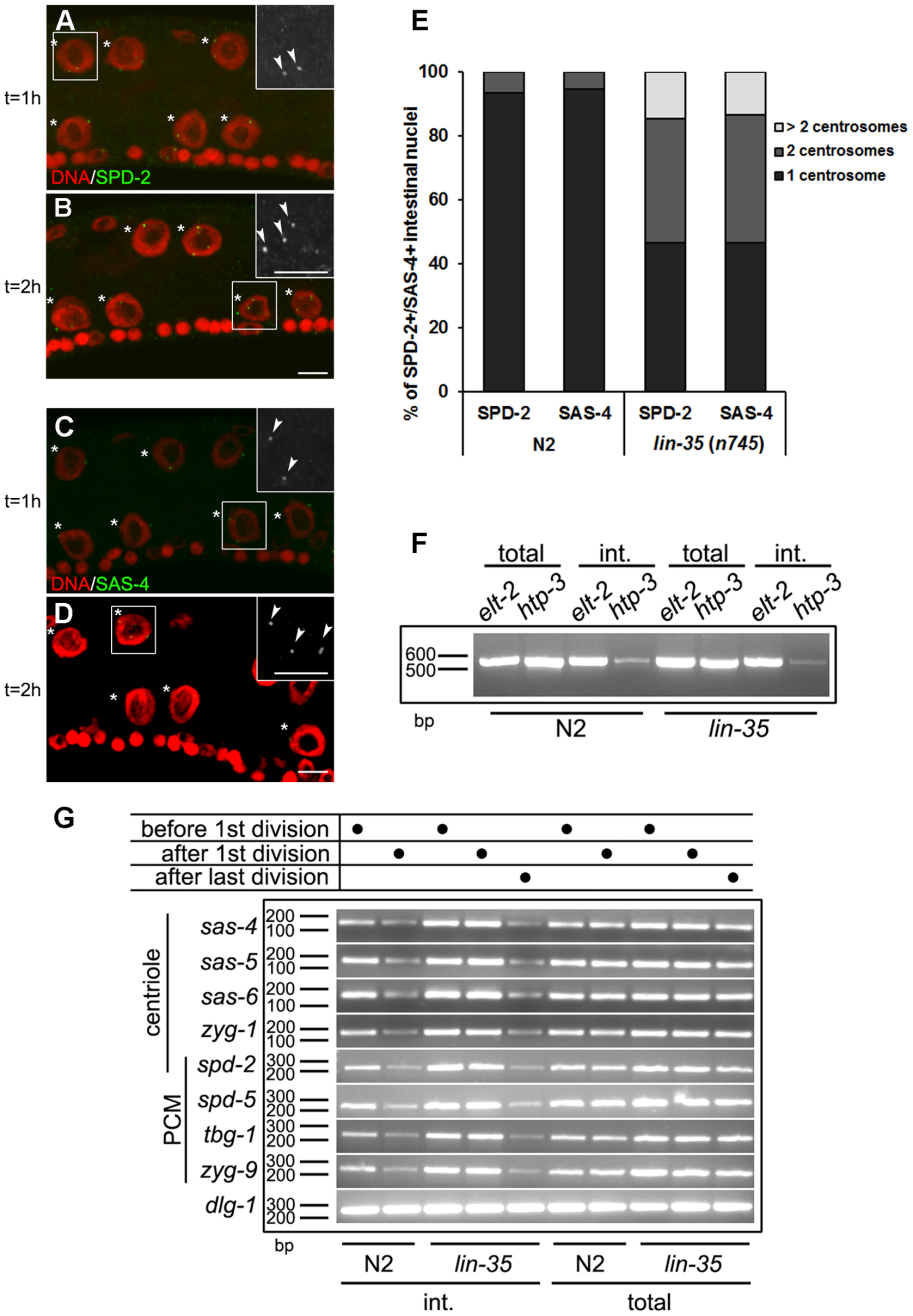
*lin-35*/Rb mutants undergo additional rounds of centriole duplication. (**A and B**) *lin-35*/Rb mutant larvae were stained with DAPI (red) and anti-SPD-2 (green) to monitor centriole dynamics at the nuclear division. The panel (A) was obtained by staining animals approximately one hour after the nuclear division (t = 1 h), while (B) was acquired two hours after the division (t = 2 h). Asterisks indicate the intestinal nuclei and the arrowheads indicate SPD-2 foci. (**C and D**) *lin-35*/Rb mutant larvae were stained with DAPI (red) and anti-SAS-4 (green) to monitor centriole numbers after the nuclear division. The insets in (A–D) represent magnified views of regions highlighted by the white rectangles. Scale bar, 5 µm. (**E**) Quantification of SPD-2 or SAS-4 foci in intestinal nuclei in both wild type and *lin-35*/Rb mutants two hours after the first intestinal nuclear division. n = 75. (**F**) RT-PCR analysis of cell-specific transcripts from N2 and *lin-35* (*n745*). *elt-2* is intestinal specific, while *htp-3* is expressed exclusively in the germ line. (**G**) The expression of *spd-2*, -*5*, *zyg-1*, -*9*, *sas-4*, *-5*, *-6*, *tbg-1* and *dlg-1* (control) [77] was quantified using RT-PCR from total or intestine-enriched mRNA from wild type (N2) or from *lin-35 (n745)* larvae before or after the first nuclear division, and 6–8 hours after the last nuclear division in *lin-35* (*n745*) mutants. int., intestinal. bp, base pair.

Since the elimination of the centrioles occurs specifically during the L2 stage, we next wondered whether elimination might be under temporal control, or whether it is part of the endocycle program and therefore contingent on the transition from the mitotic divisions to the endocycles. To distinguish between these possibilities, we used a *lin-35* (*n745*) mutant that is mutated for the *C. elegans* orthologue of Rb [43]. These animals repeat the nuclear divisions, thus giving rise to supernumerary intestinal nuclei prior to their eventual switch to the endocycle program later in L2 stage [44], but developmental timing is otherwise normal. Interestingly, although centriole numbers were unaffected in *lin-35* (*n745*) animals during the L1 stage (data not shown), two SPD-2 foci per 4C nucleus were frequently visualized in *lin-35/*Rb mutants 1-2 hours after the first nuclear division (Figure 4A, 4E, S1D). Similarly, this was reflected in the number of SAS-4 foci during the L2 stage (Figure 4C, 4E), presumably as a consequence of failing to uncouple centriole duplication from the first endo-S phase. Some animals occasionally even possessed more than two foci (Figure 4B, 4D, 4E). Because we see numeral defects in *lin-35/*Rb mutants that do not initiate the endocycle program in a timely manner, our data suggest that centriole duplication will be re-licensed as long as cells undergo a mitotic nuclear division and do not execute the endocycle program. However, following the onset of the endocycle program after the nuclear division(s) have terminated, the cells become refractory to the endo-S and will eliminate the centrioles shortly thereafter. Taken together, our data suggest that the apparent uncoupling of centriole duplication from S phase and subsequent centriole elimination relies on the initiation of the endocycle program, and does not result as a consequence of prolonged or aberrant S-phase, or chronological developmental time *per se*.

### Centriole elimination is preceded by transcriptional attenuation of genes that drive duplication

*lin-35/*Rb mutants exhibit a plethora of defects that arise due to the misexpression of genes that would normally be silenced [45]. Because we observed aberrant centriole duplication at the L1 nuclear division in the *lin-35/*Rb mutants, we reasoned that some of the misregulated gene targets in these animals might include genes involved in centriole duplication. In order to precisely analyze the expression levels of these gene products exclusively in the intestine we performed mRNA tagging to enrich for intestinal-specific transcripts following immunoprecipitation (Figure 4F) [46]. Taking advantage of this enriched fraction of intestine-specific mRNA, we monitored the levels of PCM gene products or those required for centriole duplication, at both the pre- and post L1 nuclear division.

During the L1 stage, all the known genes that affect centriole duplication were expressed at higher levels in *lin-35* (*n745*) mutants than in wild type larvae, consistent with previous analyses (Figure 4G) [45]. Elevated expression of these genes is also observed in intestinal cells, which may drive the centriole duplication during the L2 stage.

In wild type larvae, this suite of centriole duplication genes were rapidly reduced in the intestinal mRNA fraction following the nuclear division (L1/L2 transition), while their expression levels remained unchanged in mRNA obtained from whole animals (Figure 4G). This was also reflected in the expression of PCM components [37], [47]–[48]. We then compared the expression levels of these genes between wild type and *lin-35/*Rb mutants and found that they were not appropriately attenuated in the intestinal cells following the first intestinal nuclear division in *lin-35/*Rb animals (Figure 4G). However, despite their inability to downregulate these genes immediately following the nuclear division, their expression eventually drops to near wild type levels 6–8 hours after the final observed nuclear divisions take place in the *lin-35/*Rb mutants (Figure 4G). This results in a significant delay in their attenuation, similar to what is observed with the cyclin genes prior to the onset of the endocycle program [44]. These transcriptional data are further corroborated by our immunostaining results (Figure 4A–4D) and suggest that many of the key genes that regulate centriole duplication are transcriptionally attenuated at, or around, the time that the intestinal cells begin to endoreduplicate. Although this correlation is intriguing our data do not unequivocally confirm that transcriptional attenuation plays a direct role in the eventual elimination of the centrioles by the L3 stage.

### SPD-2 phosphorylation in cell cycle uncoupling and stability: CDK and PLK

Centriole duplication is controlled by many cell cycle kinases during mitotic cell division. On the other hand, kinase activities that drive G2 and M phase events are most likely reduced after the final nuclear division in the intestine which precedes centriole elimination. We therefore wondered whether any of the well-characterized cell cycle kinases might modify SPD-2 to affect this process, particularly since recent biochemical and genetic analyses have revealed that direct phosphorylation of centriolar components can impact both duplication and maturation [49]–[51].

**Figure 5:**
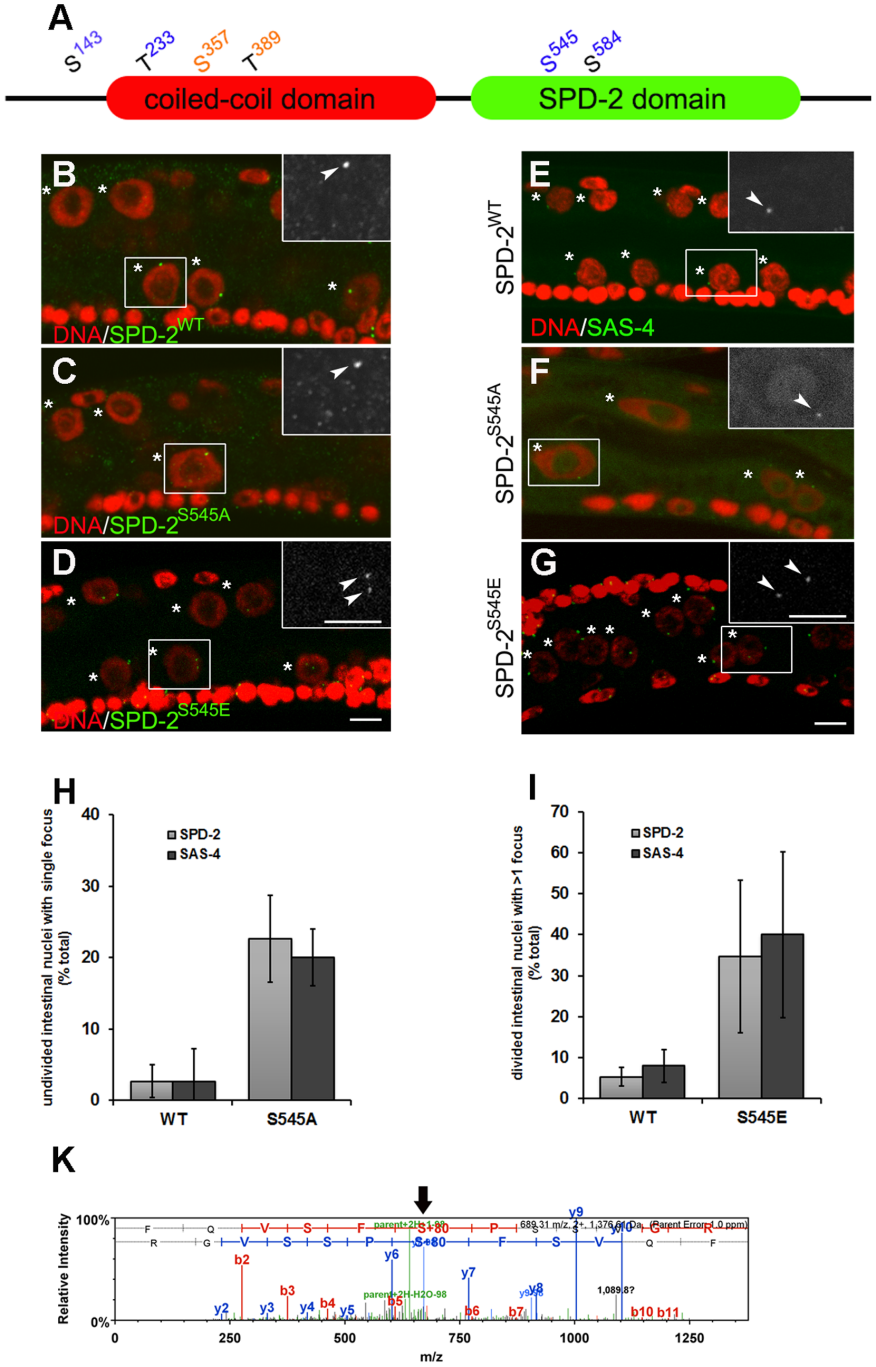
Phosphorylation of SPD-2 affects numeral regulation of centrioles in the intestinal cells. (**A**) Diagram of SPD-2 and its potential phosphorylated sites. Numbers represent amino acid position. S, Serine. T, Threonine. Orange numbers: predicted consensus PLK phosphorylation site. Blue numbers: predicted consensus CDK phosphorylation site. Blue or Orange S indicates experimentally-confirmed phosphorylated Serine [58], [78]. (**B–D**) and (**E–G**) Early L2 *spd-2* (*oj29*) animals carrying transgenic WT or the S545-variant SPD-2 following the intestinal nuclear division. DAPI (red) and SPD-2 (green) in (B–D) or SAS-4 (green) in (E–G). Asterisks indicate the intestinal nuclei and arrowheads show SPD-2 or SAS-4 foci. The insets show high magnification of the regions within the white rectangles. Scale bar, 5 µm. (**H**) The frequency of centriole duplication failure is represented by quantifying undivided intestinal nuclei associated with single SPD-2 or SAS-4 foci. (**I**) The frequency of supernumerary centriole duplication is indicated by the number of divided intestinal nuclei with more than one SPD-2 or SAS-4 focus after the nuclear division. Error bar, standard deviation; n≥75; P<0.05 (t-test). (**K**) Mass spectrometric analysis of SPD-2. +80 indicates the phosphorylated amino acid and the arrow highlights S545 in red.

Using a bioinformatic Group-based Prediction System (GPS) [52]–[53], we identified a number of predicted phosphorylation sites on SPD-2 (Figure 5A). In order to experimentally test whether these amino acids affect centriole fate in the endocycling intestinal cells we mutated each of these sites to either Alanine (A) or Glutamic Acid (E) to convert the wild type SPD-2 sites into non-phosphorylable or phosphomimetic variants, respectively. The variants, or the wild type SPD-2, were integrated as single copies into the genome [54] and the resulting transgenic animals were crossed into the *spd-2* (*oj29*) mutant background [55].

We found that the wild type transgene (SPD-2^WT^) or the SPD-2 variants could rescue *spd-2* (*oj29*)-associated embryonic lethality at 25°C from 100% to less than 4.5% (n≥110), suggesting that these transgenes possess essential SPD-2 function. However, among all the variants, only SPD-2^S545A^ transgenic animals, where a predicted CDK-targeted Serine residue at 545 was mutated to Alanine, demonstrated apparent defects whereby the intestinal nuclei frequently failed to divide at the L1/L2 transition (Figure 5C), compared to SPD-2^WT^ animals (Figure 5B). We monitored centriole numbers in the SPD-2^S545A^ variants at the time of the nuclear division and found that only one SPD-2 focus was detectable adjacent to the undivided nuclei at the L2 stage (Figure 5C). The absence of centriole duplication is not due to the lack of S phase in these intestinal cells since they possess twice the DNA content of their divided neighboring intestinal nuclei (Figure S1E, C value 4.27 vs. average 2.11), suggesting a problem in centriole duplication. This occurred in approximately 25% of the intestinal nuclei in SPD-2^S545A^ animals (Figure 5H). Again, we observed similar defects in centriole duplication when we monitored SAS-4 levels in the SPD-2^S545A^ background (Figure 5E, 5F). A single SAS-4 focus (1 centriole pair) was frequently observed adjacent to a failed nuclear division in the L2 stage (Figure 5H), indicating that the disruption of SPD-2 phosphorylation on S545 compromises centriole duplication and renders the intestinal nuclei incapable of dividing as they transition into the L2 and the associated endocycle program.

CDK-2 activity, paired with either cyclin E or cyclin A, plays a central role in centriole duplication in many species [5]. The defect in centriole duplication in the SPD-2^S545A^ variants led us to further examine if CDK-2 is required for centriole duplication in the intestine during the L1. We performed *cdk-2*(*RNAi*) using an intestine-specific RNAi-sensitive strain circumventing any essential function of the gene during development [56]-[57]. Consistent with the centriole duplication defects in the SPD-2^S545A^ variants, less than 20% of the intestinal *cdk-2*(*RNAi*) animals had duplicated their centrioles after 13 h into the L1 stage (Figure S4B, S4C), while nearly 70% of the intestinal nuclei were associated with two SPD-2 foci in control animals (Figure S4A, S4C), suggesting that SPD-2^S545A^ may be an important CDK-2 target in regulating timely centriole duplication.

Conversely, 35% of the SPD-2^S545E^ post-division nuclei were associated with the appearance of supernumerary SPD-2 foci in the intestinal cells during the L2 stage (Figure 5D, 5I), whereas centriole duplication was not noticeably affected during the L1 stage (data not shown). Moreover, following the intestinal nuclear division, the C value of intestinal nuclei range from 4.08 to 4.14 in mid-L2 stage (Figure S1F; n = 20; p>0.1), suggesting that intestinal nuclei divide normally in the phosphomimetic SPD-2^S545E^ variants and the supernumerary SPD-2 foci appear during the L2. Moreover SPD-2^S545E^ was not sufficient to rescue the centriole duplication phenotype in *cdk-2*(*RNAi*) animals indicating that S545 is most probably not the only critical CDK2 target on SPD-2 (data not shown). Supernumerary SAS-4 foci were also detectable in the SPD-2^S545E^ variants suggesting that this single amino acid change does not uniquely affect SPD-2 levels and or function, but rather its effects impinge on the entire centriole (Figure 5G, 5I). This phenotype is distinct from the *lin-35/*Rb mutant since the intestinal nuclei do not undergo additional divisions, indicating that SPD-2^S545E^ specifically affects the numeral control by affecting centriole duplication without impinging directly on cell cycle progress.

Mass spectrometric analysis confirmed that these residues were indeed phosphorylated *in vivo* in the intestinal cells at the time of the L1/L2 transition (Figure 5K). We also found using *in vitro* kinase assays that S545 could be phosphorylated by human CDK-2/cyclin A and CDK-1/cyclin B (Figure S4D, S4E), but not by CDK-2/cyclin E (data not shown). Taken together, S545 is likely to be a physiologically relevant CDK target site on SPD-2 that affects centriole duplication.

### The SPD-2^S357^ phosphomimetic variant stabilizes SPD-2 and affects centriole stability in the endocycling intestinal cells

**Figure 6:**
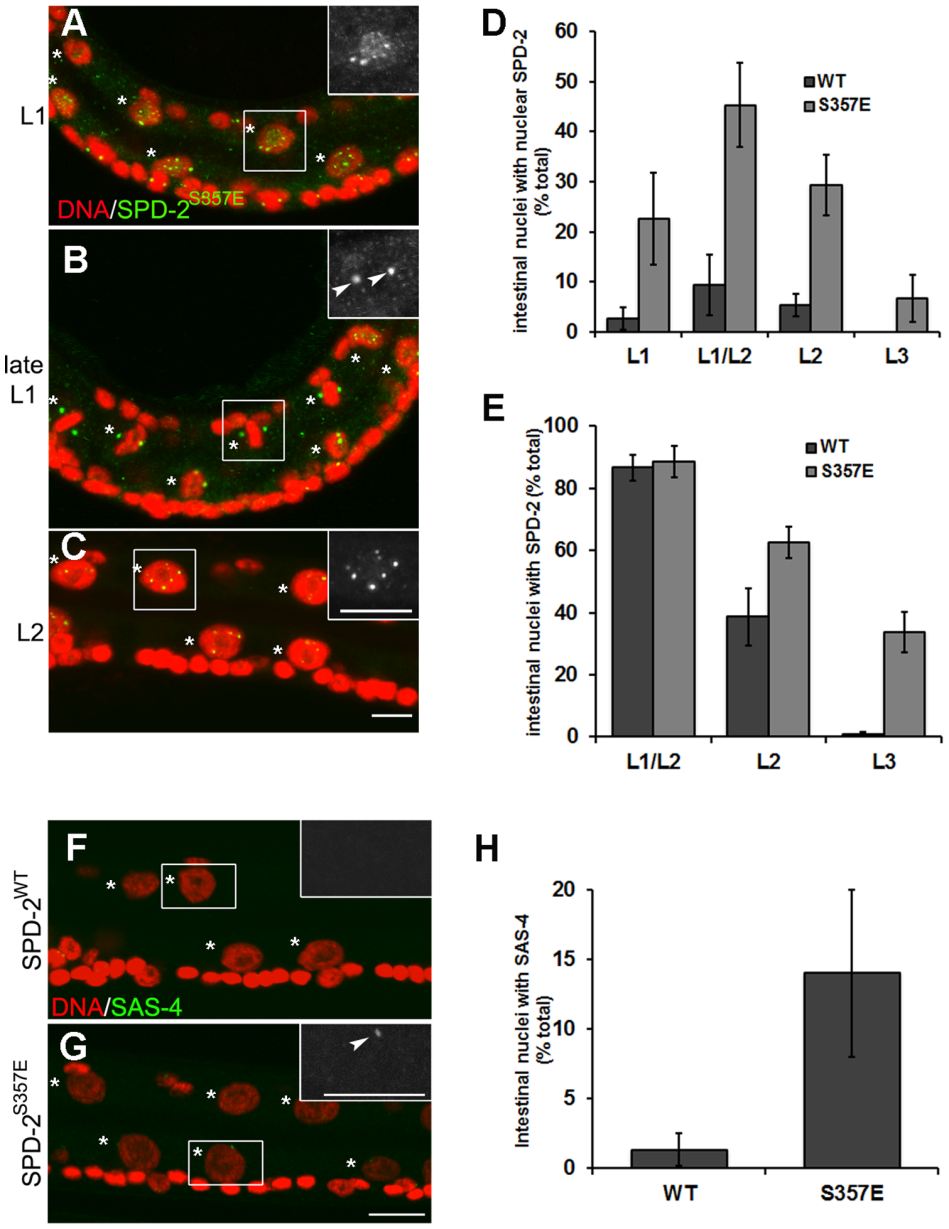
Phosphorylation of S357 on SPD-2 affects appropriate localization and stability. (**A–C**) *spd-2* (*oj29*) animals carrying the SPD-2^S357E^ variant were stained with DAPI (red) and anti-SPD-2 (green) in the L1, L1/L2 and L2. The asterisks indicate the intestinal nuclei. Scale bar, 5 µm. (**D**) SPD-2 staining was monitored in intestinal cells and the percentage of intestinal nuclei that demonstrate nuclear-localized SPD-2 in strains carrying either wild type SPD-2 or SPD-2^S357E^ variant were determined. (**E**) The frequency of SPD-2 persistence is quantified by counting the number of intestinal cells that continue to show any SPD-2 signal at later larval stages. Error bar, standard deviation; n≥50; P<0.05 (t-test). (**F**) Late L2 *spd-2* (*oj29*) animals expressing the SPD-2^WT^ or (**G**) the SPD-2^S357E^ variant were stained with DAPI (red) and anti-SAS-4 (green). The number of SAS-4 foci were quantified and indicated in (**H**). The asterisks indicate the intestinal nuclei while arrowheads show SAS-4 foci. Error bar, standard deviation; n = 50; P<0.05 (t-test). Scale bar, 5 µm.

SPD-2 was previously reported to be phosphorylated on a consensus PLK phosphorylation site at Serine S357 [58]. We also identified S357 in our MS/MS analysis following an *in vitro* kinase assay performed with human PLK-1 and GST::SPD-2, although we were unable to detect the phosphorylation on S357 *in vivo* (Figure S5A and data not shown). Consistent with a functional role for this site, the phosphomimetic modification of Serine S357 (SPD-2^S357E^) caused SPD-2 to accumulate in aggregate-like puncta, paralleled by an increased frequency of cells with nuclear-localized SPD-2 prior to the nuclear division (Figure 6A, 6D), whereas the nuclear localization of wild type SPD-2 usually precedes its elimination during the L2 stage (Figure 2D). This SPD-2 accumulation has no apparent effect on centriole function in MTOC, since SPD-2 is normally present on the opposing poles of the condensing nuclei prior to nuclear division (Figure 6B). SPD-2^S357E^ is still detectable in a number of intestinal cells even by the end of L2, long after SPD-2^WT^ normally disappears (Figure 6C, 6E). This persistence of SPD-2^S357E^ was not always reflected by the SAS-4 levels, but nonetheless 14% of the SPD-2^S357E^ variant intestinal cells still possessed SAS-4 foci in the late L2 stage (Figure 6F-6H), suggesting that a portion of the centrioles are stabilized in this variant background. Therefore the phosphomimetic SPD-2^S357E^ modification is sufficient to stabilize some SPD-2, causing it to accumulate in aggregate-like nuclear puncta. The same modification of SPD-2 also delays the elimination of some, but not all centrioles based on the persistence of the centriolar marker SAS-4.

In order to confirm that PLK-1 might stabilize SPD-2 in these cells we monitored the levels of SPD-2 at the L1/L2 transition using intestinal-specific *plk-1*(*RNAi*) animals. In these animals, despite that two SPD-2 foci are present, about 15% of the intestinal nuclei fail to divide (Figure S5B–S5D), suggesting that PLK-1 is required for the intestinal nuclear division, but not centriole duplication. Moreover, SPD-2 was not abnormally destabilized in *plk-1*(*RNAi*) animals, or in the non-phosphorylable SPD-2^S357A^ variants, suggesting other factors may regulate the stability of SPD-2 in addition to PLK-1.

### Ubiquitin mediated degradation of SPD-2

**Figure 7:**
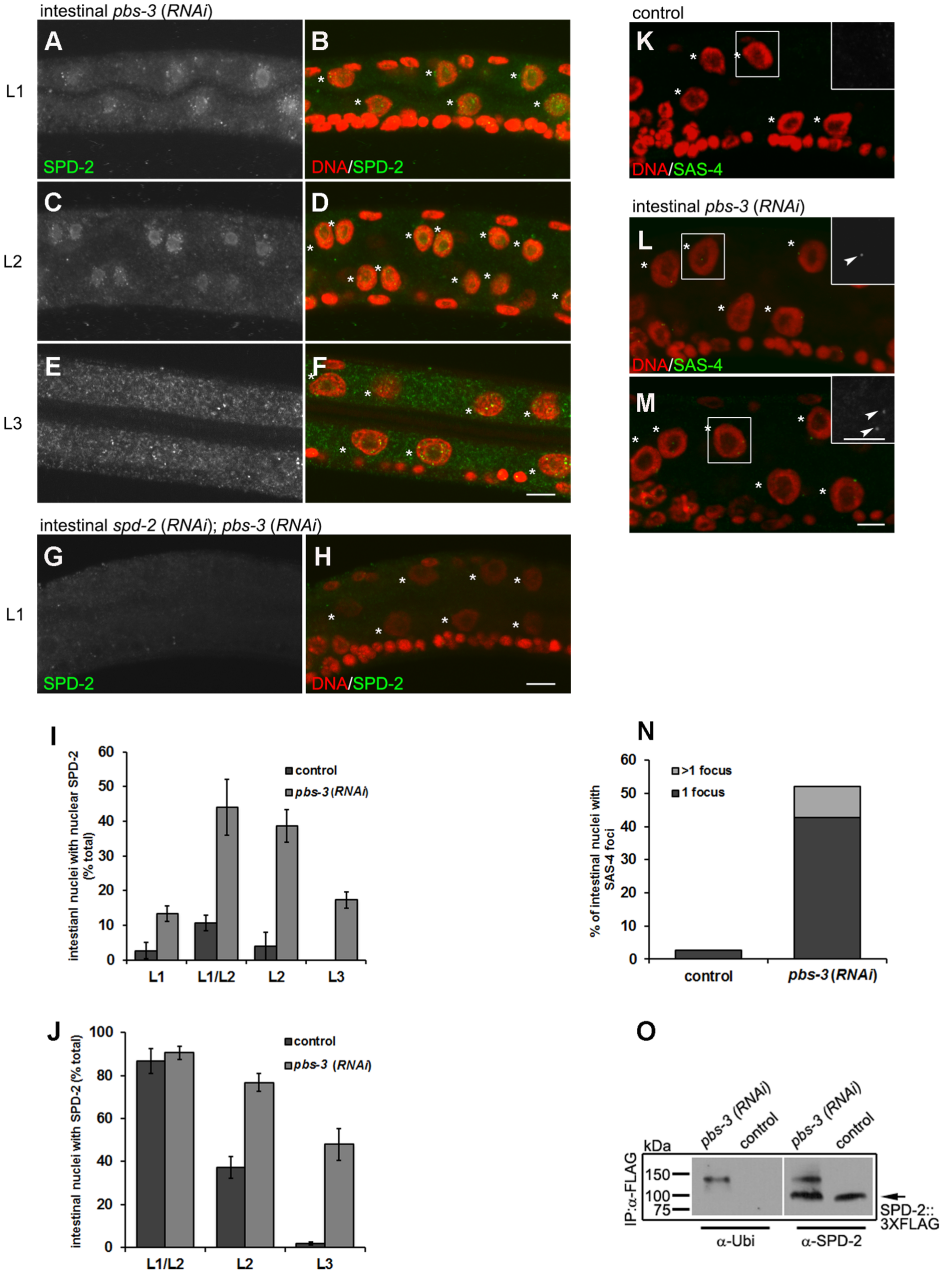
SPD-2 is ubiquitylated and its localization and stability are dependent on proteasome function. (**A–F**) Larvae were subjected to *pbs-3*(*RNAi*) and subsequently stained with DAPI (red) and anti-SPD-2 (green) in the L1, L2 and L3 stages, respectively. (A), (C) and (E) show anti-SPD-2 alone. (**G–H**) Larvae were subjected to *pbs-3*(*RNAi*); *spd-2*(*RNAi*) and subsequently stained with DAPI (red) and anti-SPD-2 (green) in the L1. (G) shows anti-SPD-2 alone. Asterisks indicate the intestinal nuclei. Scale bar, 5 µm. (**I**) SPD-2 nuclear localization was monitored and the number of intestinal cells that demonstrate diffuse nuclear SPD-2 staining was compared in control and *pbs-3*(*RNAi*). (**J**) The effects of *pbs-3*(*RNAi*) on SPD-2 stability were quantified by determining the number of intestinal cells that express SPD-2 at later larval stages. Error bar, standard deviation; n≥50; P<0.05 (t-test). (**K–M**) SAS-4 levels were stained in the intestinal cells of late L2 *pbs-3*(*RNAi*) animals and were quantified as above (**N**). The asterisks indicate the intestinal nuclei, while arrowheads point to SAS-4 foci. Scale bar, 5 µm. n≥50; P<0.05 (t-test). (**O**) Homogenates obtained from animals expressing 3XFLAG tagged SPD-2 subjected to *pbs-3*(*RNAi*) or *gfp*(*RNAi*) (control), were incubated with anti-FLAG antisera and associated proteins were immunoprecipitated with Protein A-agarose. The pellets were blotted with anti-ubiquitin or anti-SPD-2 respectively. kDa, kilo Dalton.

Recent data have implicated ubiquitin-mediated proteolytic degradation in the appropriate regulation of centrosomal components [59]–[61]. If SPD-2 abundance is controlled by ubiquitin-mediated degradation then the absence of essential ubiquitylation/proteasome components may also affect the elimination of the intestinal centrioles. *proteasome b-subunit* 3 (*pbs-3*) is essential for proteasome function [62] and in *pbs-3*(*RNAi*) animals, SPD-2 shows substantial nuclear accumulation (Figure 7A–7D, 7I), while SPD-2 signal is still detectable in the L3 stage, considerably later than in control animals (Figure 7E, 7F, 7J). Both the accumulation and persistence of SPD-2 are greatly reduced in *pbs-3*(*RNAi*); *spd-2*(*RNAi*) animals, confirming that the signals are indeed SPD-2-specific (Figure 7G, 7H). Similar effects on SPD-2 were also observed in *pbs-5*(*RNAi*) animals alone (data not shown), consistent with a role of proteasome-mediated degradation in the timely elimination of the centrioles after the intestinal cells commence endoreduplication. Intriguingly, in addition to the delayed centriole elimination phenotype, anti-SAS-4 staining revealed that centriole duplication was occasionally observed in *pbs-3*(*RNAi*) animals (Figure 7K–7N), which could reflect the stabilization of additional proteins involved in centriole duplication, or other key effectors of numeral regulation. Furthermore, by compromising proteasome function we were able to detect a higher molecular weight band in our FLAG-tagged SPD-2 immunoprecipitates. This band was recognized by both anti-SPD-2 and anti-Ubiquitin, consistent with the ubiquitylation of SPD-2 *in vivo*, which likely precedes its degradation (Figure 7O).

Overall, our data suggest that during the switch to the endocycle program SPD-2 is ubiquitylated and subsequently degraded by the proteasome. Mimicking the potential PLK-1-dependent phosphorylation on S357 enhances nuclear localization and aggregation of SPD-2, which may interfere with destabilizing modifications that signal the removal of SPD-2. However, other cues that over-ride this modification must occur after the nuclear division to destabilize the centriole-associated SPD-2 and other centriolar components during the elimination process.

## Discussion

The intimate links between the cell division cycle and the centrosome cycle ensure that the duplication and maturation of the centrosome occur in synchrony with the formation of the central spindle during mitosis. Consistent with this, many of the key enzymatic activities that drive the events specific to each stage of the cell cycle simultaneously affect aspects of the centrosome cycle [63].

**Figure 8:**
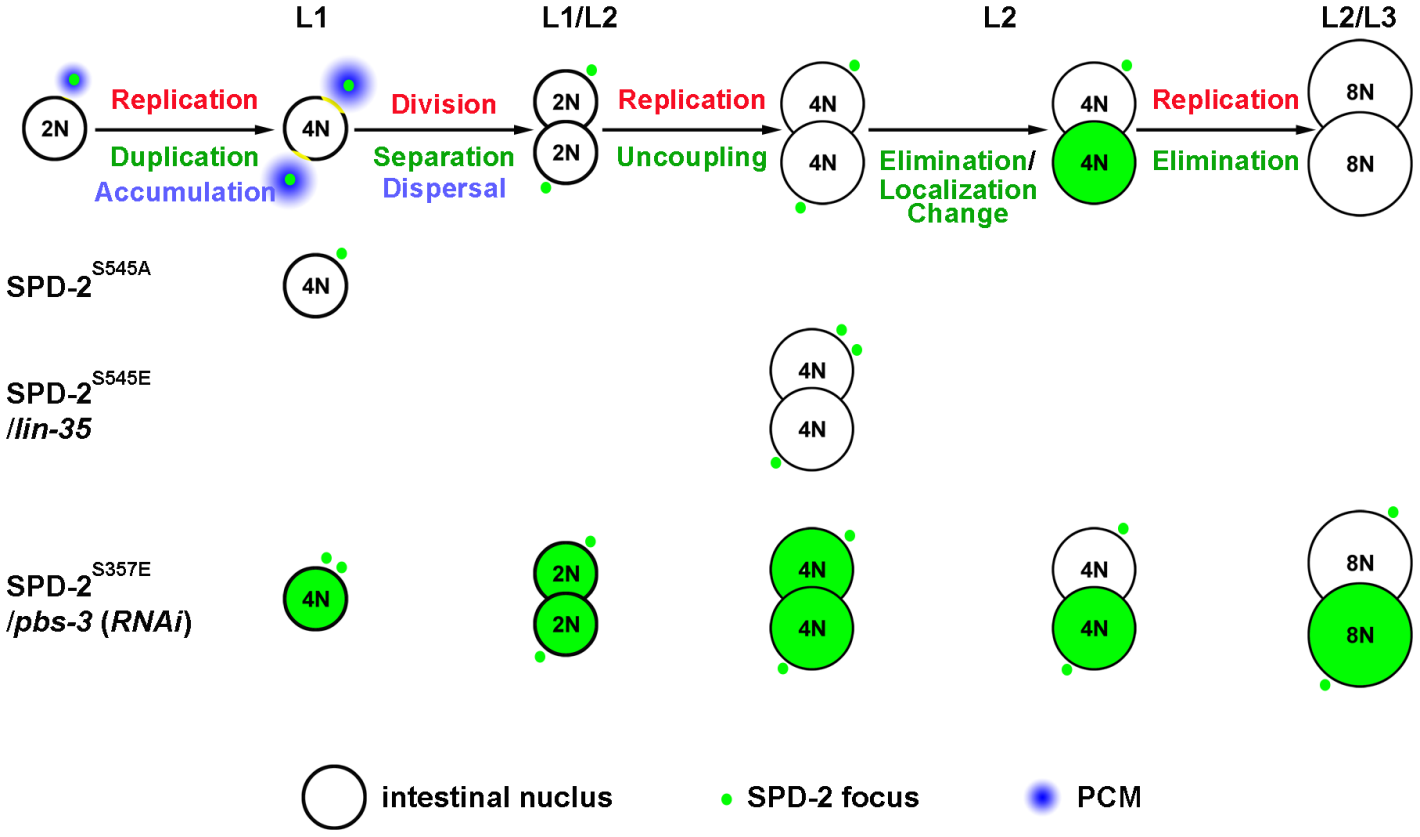
A model to depict centriole uncoupling and elimination in the postembryonic *C. elegans* intestine. The complete postembryonic lineage of single intestinal cell in the wide type animals is shown. Each SPD-2 focus (green) represents a centriole pair. Centrioles undergo duplication and separation during the L1 nuclear division. The centrioles then lose their PCM during anaphase and undergo cell cycle “uncoupling” whereby centrioles are unaffected by the oscillations of successive endocycle S-phases. These events precede the diffusion of SPD-2 into the intestinal nuclei followed by its eventual elimination. The effects of SPD-2 variants or genetic backgrounds on centriole duplication/stability are highlighted at the relevant developmental stages. Substituting Serine 545 with Alanine on SPD-2 results in centriole duplication failure, whereas replacing Serine 545 with Glutamic Acid or, alternatively in *lin-35* mutants, centrioles overduplicate. SPD-2 becomes stabilized when *pbs-3* is abrogated or if SPD-2 S357 is converted to a phosphomimetic residue.

During the development of many organisms however, the mitotic cell cycle is replaced by the endocycle: an alternative means to provide tissue mass or to increase nuclear output/volume [32]. We were interested in how centrioles would respond to this modified cell cycle. We found that the centrioles appear to undergo a cell cycle uncoupling event shortly after the intestinal nuclear division. This uncoupling is contingent on the transition from mitotic cell division to an endocycle program at the end of the L1 stage. Following this division the centrioles are no longer duplicated and are eliminated during the L2 stage; an event that is an intrinsic developmental fate in these endocycling cells (Figure 8).

In cycling cells, the numeral integrity of centriole numbers is maintained through the regulation of centriole duplication driven by various cell cycle kinases including CDK2, in addition to a licensing mechanism that restrains the duplicated centrioles from responding to S-phase activities until after anaphase [42], [64]. Recent observations suggest that alternative mechanisms may also contribute to the numeral integrity and may be context-specific [65]–[66]. Although we cannot formally exclude that the centrioles do indeed duplicate during the endo-S but simply cannot be resolved due to their proximity or tight engagement, that we were able to observe supernumerary centrioles in the SPD-2^S545E^ variant would suggest that this residue may be a target of the licensing mechanism *per se* in the intestinal cells. It is further tempting to implicate CDK activity in this step since both CDK1 and CDK2 can phosphorylate this residue *in vitro*. When the intestinal cells terminate the nuclear division, this residue may be unphosphorylated and persist in that state in the resulting daughter nuclei that will never execute a typical G2 or M-phase after that point. This modification could potentially contribute to licensing centriole duplication in the daughter cells.

γ-tubulin, a key PCM component that also plays important roles in centriole duplication [22], undergoes rapid anaphase dispersal from the centrosome at the end of the L1 stage in intestinal cells. Interestingly, centriole duplication still occurs in the SPD-2^S545E^ variant after this point, perhaps because in *C. elegans* γ-tubulin is not essential for the initial recruitment of SAS-4 to the centriole [33], a crucial step for centriole duplication. Consistent with this, ultra-structural studies in *C. elegans* revealed that reducing γ-tubulin may result in abnormal centriole structure but does not necessarily block duplication [67]. At this point we cannot rule out that the ultra-structure of the supernumerary centrioles in our SPD-2^S545E^ variants is not altered, nor can we exclude that the SPD-2^S545E^ may recruit SAS-4 more efficiently so that the elevated levels of SAS-4 can compensate for the subthreshold levels of cytoplasmic γ-tubulin.

Centrosome/cell cycle uncoupling is not unique to endocycling cells as this occurs in other developmental contexts as well. During spermatogenesis in several different organisms the haploid sperm fertilizes the oocyte with a pair of centrioles, indicating that centriole duplication had taken place during meiosis II in the absence of DNA replication [68]. Similarly, in the multiciliated cells of the trachea, many centrioles arise spontaneously, independent of cell division [26]. In *C. elegans*, centrosome/cell cycle uncoupling also occurs in the endocycling anterior daughter of the V cells, but the effects we observed with the SPD-2^S545E^ variants are restricted to the centrioles of the intestinal cells. How the centriole is generally sensitized to the various activities associated with cell cycle progression, or how it duplicates independently of these influences may be entirely cell-type specific.

The final nuclear division is a critical decision point, after which the centrioles become insensitive to the first endocycle S-phase and are eliminated shortly thereafter. Their eventual elimination is not dependent on their state of licensing nor a response to aberrant S phase activity, but rather a defined developmentally-controlled process typical of the intestinal endocycle program.

The PLK-1 site may be important to block centriole turnover in that the phosphomimetic SPD-2^S357E^ replacement stabilizes SPD-2 and enhances its aggregation (Figure 8). Our data suggest however that the centrioles are not destabilized in the non-phosphorylable SPD-2^S357A^ variant or in *plk-1*(*RNAi*) animals, indicating that additional mechanisms must function redundantly with PLK-1 to regulate centriole stability. How this phosphorylation affects stability is still questionable, but it may affect recognition by the proteasomal degradation pathway, since this pathway plays a key role in the timely elimination of the centrioles, not only in the intestinal endocycles, but also in mammalian cells and *Drosophila* [59], [61] (Figure 8),

It is tempting to generalize a role for of SPD-2 in all developmental contexts that might include uncoupling and/or elimination. In germ cells that initiate oogenesis, SPD-2 also changes its cellular distribution prior to the elimination of the centrioles in leptotene (Figure. S2). However, in the germ line, modification of S357 on SPD-2 appears to have little or no consequence. Moreover, gene products that have been identified to affect the timing of centriole elimination in the germ line [36] have no effect on centrosome/cell cycle uncoupling and/or elimination in the gut (data not shown). Despite the differences between the two tissues, the pathways may nevertheless converge upon a common component, SPD-2, where both modes of regulation independently impinge on the cellular SPD-2 distribution to ultimately affect its stability.

Although centriole elimination during oogenesis is common in many organisms, it also occurs in various other contexts, namely after the cell commits to a terminally differentiated state. In most mammalian cells the centriole pair will undergo specific modifications to generate a basal body to contribute to the primary cilium following the last mitotic division [69], while in *Drosophila* and *C. elegans* this is not the case, and only a small collection of cells are ciliated [70]–[71]. Interestingly, the epithelial cells of *C. elegans*, such as the seam cells (Figure 1J–1K′, 1L, 1M) and the vulva cells (Figure 1N–1Q), do not possess primary cilia. In these cells SPD-2::GFP remains visible until they finish their final division, suggesting that SPD-2 may also be targeted for elimination upon terminal differentiation. It is not clear why it would be advantageous to remove the centrioles from a differentiated cell. Perhaps its presence could sensitize the cell to regenerate the mitotic spindle through some aberrant acquisition of MTOC capacity, potentially a critical step toward unscheduled/unequivalent divisions that could drive hyper- or neoplasia. In this light, in addition to its co-opted role in signaling, the role of the primary cilium may be to sequester the centriole for future entry into the mitotic cell cycle, such as during wound healing for example. Our interrogation of how such organelles are removed in a developmentally-regulated manner will be informative to identify genes that are generally required to control this process in a coordinated manner in diverse cell types.

## Materials and Methods

### Strains and alleles

*C. elegans* Bristol strain N2 was used as the wild-type strain and was cultured as described previously [72]. Strains carrying a single copy of tagged wild type SPD-2, or variants thereof were generated as described [54]. L1/L2 transition is defined with the intestinal nuclear division, whereas L2/L3 with the larval molt. All strains with the temperature sensitive *spd-2* (*oj29*) allele were maintained at the permissive temperature and all experiments were performed on larvae grown previously for 6 h at 25°C. The following alleles were used in this study: *spd-2* (*oj29*), *lin-35* (*n745*) and *cul-4* (*gk434*). MR0156 (*rrIS01*[elt-2::GFP]X), MR1567 (*rrIS1567*[*spd-2*::SPD-2::3XFLAG; *unc-119*(+)] II; *unc-119*(*ed3*)III), MR1657 (*unc-13* [*e1091*] *spd-2*[*oj29*] I; (*rrIS1495* [*spd-2*::SPD-2::GFP; *unc-119*(+)]II; *unc-119*(*ed3*)III), MR1672 (*unc-13*[*e1091*] *spd-2*[*oj29*] I; (*rrIS1488* [*spd-2*::SPD-2(S545A)::GFP; *unc-119*(+)]II; *unc-119*(*ed3*)III), MR1667 (*unc-13*[*e1091*] *spd-2*[*oj29*] I; (*rrIS1514* [*spd-2*::SPD-2(S545E)::GFP; *unc-119*(+)]II; *unc-119*(*ed3*)III), MR1652 (*unc-13*[*e1091*] *spd-2*[*oj29*] I; (*rrIS1587* [*spd-2*::SPD-2(S357E)::GFP; *unc-119*(+)]II; *unc-119*(*ed3*)III), MR1778 (rrIS1778 [*elt-2*::FLAG::PAB-1; *unc-119*(+)]II; *unc-119*(*ed3*)III), MR1779 (*lin-35*(*n745*)I; rrIS1778 [*elt-2*::FLAG::PAB-1; *unc-119*(+)]II; *unc-119*(*ed3*)III), MR1785 (*rrEx1785* [*elt-2*::RDE-1; *inx-6*::GFP]; *rde-1*(*ne219*)V).

### DNA constructs, site-directed mutagenesis, RNAi and HU treatment

For the transgenes encoding either the wild type or the GFP-tagged SPD-2 variant, a 2433 bp sequence upstream of the full length *spd-2* but lacking its natural stop codon was first amplified from N2 and cloned in frame upstream of GFP in pPD95.79 following digestion with SalI and XmaI pMR812. Subsequently a fragment containing *spd-2*::SPD-2::GFP::*unc-54* 3′UTR was removed from pMR812 and then cloned into pCFJ151 to yield pMR831. PCR for site-directed mutagenesis was performed using Gene-Tailor site-directed mutagenesis (Invitrogen) on pMR831 in order to generate SPD-2 variants including pMR832 (S545A), pRM833 (S545E) and pMR857 (S357E).

The 3XFLAG including a stop codon was first cloned into pPD49.26 to generate pMR837. Subsequently a 2433 bp sequence upstream to the start codon of *spd-2* and the genomic DNA encoding full length *spd-2* lacking its natural stop codon was amplified and cloned in frame into pMR837 so that 3XFLAG would be C-terminal thus generating pMR841. Eventually, a *spd-2*::SPD-2::3XFLAG::*unc-54* 3′UTR was removed from pMR837 and then cloned into pCFJ151 to yield pMR850.

For the intestinal mRNA enrichment experiment, the *elt-2* promoter and FLAG::PAB-1 [46], were flanked with sequences compatible for Gateway cloning (Invitrogen) and cloned into pCFJ150 to generate pMR869. For tissue-specific RNAi, the *elt-2*::RDE-1::*unc-54* 3′UTR was cloned into pMR542. For recombinant GST::SPD-2, *spd-2* cDNA was cloned into pGEX-6p-2 to generate pMR873 (GE Healthcare).

The *cdk-2*(*RNAi*), *plk-1*(*RNAi*) and *pbs-3*(*RNAi*) presented in the microscopy were performed with our tissue-specific RNAi system, whereas the *pbs-3*(*RNAi*) presented in the western blot (Figure 7O) was performed by conventional RNAi using the Ahringer feeding RNAi library [62].

HU treatment was performed as described elsewhere [73]. For the HU block of the final mitotic intestinal cell cycle, synchronized L1 animals were cultured under standard conditions for five hours [72], followed by a 6-hour HU treatment. DNA content was unchanged over the course of our experiment indicating the HU block was efficient. For the HU block in the endoreduplicating intestinal cells, animals were allowed to develop under standard conditions until the completion of intestinal nuclei division [72] and then treated with HU for six hours. Centrioles and DNA content were monitored accordingly.

### Microscopy

With the exception of Figures 1J–1K′, 1N–1O, 2A and S2, other indirect microscopy was performed using a 63× oil-immersion objective lens on a LSM510 META Confocal microscope (Zeiss) 1–4 µm optical sections were acquired with a 109.6 µm pinhole and 1024×1024 pixel resolution. For each experiment, intestinal nuclei samples were taken from no less than 15 different animals to avoid any bias. Focal planes containing centrioles were projected using an LSM510 Version 3.2 SP2. Images were processed using Adobe Photoshop, preserving relative image intensities within a series. The microscopic work of Figure S2 was performed using 100× oil-immersion objective lens in a DeltaVision Image Restoration System (Applied Precision). Data were collected as a series of 13–27 optical sections in increments of 0.2 µm under standard parameters with the softWoRx 3.0 software (Applied Precision). The microscopic work of Figure 1J–1K′, 1N-1O, and 2A were performed using 63× oil-immersion objective lens in a Zeiss AX10 microscopy under stander parameters with the AxioVision 4.8 software (Zeiss). All microscopies were performed at 20°C.

### Purification of GST::SPD-2 and *in vitro* Kinase Assay

SPD-2 was overexpressed as a N-terminal glutathione-S-transferase (GST) fusion protein in BL21 bacteria following a 6-hour IPTG-induction at 37°C. Protein was purified with Glutathione Sepharose 4B (GE Healthcare) and 50 µl of eluted GST::SPD-2 fusion protein was incubated with human CDK2/cyclin E, CDK2/cyclin A, CDK1/cyclin B, or PLK-1 (Millipore) respectively for 30 minutes. Reactions were subjected to 8% SDS-PAGE and the corresponding bands were excised and analyzed by MS.

### PI staining, Antibodies and Immunological methods

#### PI staining

PI (Sigma P-4170) staining and the measurement of DNA content were performed as described elsewhere [34].

#### Immunofluorescence

The following primary antibodies and dilutions were used: 1∶100 anti-SPD-2 rabbit polyclonal [74] (a gift from Dr. K. O'Connell, National Institutes of Health, Bethesda, MD), 1∶100 anti-SAS-4 rabbit or goat polyclonal (Santa Cruz Biotechnology, SC98949 and SC20418), 1∶100 rabbit anti-γ-tubulin (Sigma-Aldrich, T1450), 1∶100 mouse monoclonal anti-α-tubulin (Sigma-Aldrich T9026) and 1∶100 mouse monoclonal anti-GFP (Abcam, ab1218). 1∶250 secondary antibodies were anti-mouse or anti-rabbit AlexaFluor 488 and anti-rabbit AlexaFluor 555 (Molecular Probe). DAPI (Sigma-Aldrich) was used to reveal DNA. L1, L2 or L3 larvae were fixed and stained as described previously [75].

#### Immunoprecipitation, purification and Immunoblotting

To confirm *in vivo* phosphorylation, 30 ml of packed young larvae were collected and re-suspended in one volume of lysis buffer [20]. Animals were lysed using repetitive 15-second sonication for 15 cycles on ice with 30-second intervals between each cycle. The lysate was then centrifuged at 4°C for 10 minutes. Supernatants were incubated with 250 µl anti-FLAG affinity gel (Sigma Aldrich) for 2 hours at 4°C. The gel was washed with lysis buffer and the beads were resuspended in 0.1 M Glycine buffer (pH2.5), centrifuged, and 50 µl of the eluate was subjected to 8% SDS-PAGE, followed by coomassie blue staining described elsewhere (http://www.mass-spec.siu.edu/CoomassieStainingProtocol). The pieces of polyacrylamide gel corresponding to SPD-2::3XFLAG predicted size were cut for MS analysis.

To detect SPD-2 ubiquitylation, proteins were purified from 10 ml of packed worms of each genotype as described above. 40 µl of the eluate was then subjected to 8% SDS-PAGE then transferred to a nitrocellulose membrane (Bio-Rad) and blotted as described elsewhere: (http://www.cellsignal.com/support/protocols/Western_BSA.html).

The following primary and secondary antibodies were used: 1∶1000 anti-SPD-2 rabbit polyclonal, 1∶500 anti-Ubiquitin mouse monoclonal (Santa Cruz Biotechnology, SC-8017), and 1∶2500 goat anti-rabbit or mouse HRP conjugated IgG (Bio-Rad). Protein bands were detected using chemifluoresence (Clarity Western ECL Substrate, Bio-Rad).

### Intestinal mRNA enrichment and RT-PCR

The intestinal mRNA tagging protocol was described elsewhere [46]. The amount of RNA from each sample was calibrated to generate comparable amplicon levels for the control genes *dlg-1* and *glo*-1. PCR was performed for 15 cycles with ProtoScript M-MuLV *Taq* RT-PCR Kit (NEB, E6400S) and gene-specific primers for each query gene. The primers were designed and analyzed with on-line tool (www.basic.northwestern.edu/biotools/oligocalc.html). Two pairs of primers were designed for each tested gene except for *elt-2* and *htp-3* (one pair). The primer sequences are listed in the Form S1.

## Supporting Information

Figure S1 **The coupling of centriole duplication with S-phase is regulated developmentally and can be genetically altered.** (**A–C**) *spd-2* (*oj29*) animals carrying the wild type SPD-2::GFP were stained with PI (Red) in the L1 and L2. (**D**) *lin-35* (*n745*) animals were stained with PI (red) and anti-SPD-2 (green) in the L2. (**E**) *spd-2* (*oj29*) animals carrying the SPD-2^S545A^ variant were stained with PI (Red) at the L1/L2 transition. (**F**) *spd-2* (*oj29*) animals carrying the SPD-2^S545E^ variant were stained with PI (Red) in mid-L2 stage. The asterisks indicate the intestinal nuclei and the arrow heads point out the SPD-2 foci. The arrows indicate the muscle cells that are used as the 2C reference and the numbers under the intestinal nuclei indicates their DNA content. Scale bar, 5 µm.
(TIF)

Figure S2 **SPD-2 diffuses into nuclei before its elimination in the germ line.** The gonad of a N2 young adult was dissected and stained with DAPI (red) and SPD-2 (green). Scale bar, 5 µm.
(TIF)

Figure S3 **γ-tubulin dispersal from the centriole affects its MTOC function.** (**A**) N2 at late L2 were stained with DAPI (blue), anti-SAS-4 (red) and anti-γ-tubulin (green). (a) highlights a metaphase nucleus while the lineage bracket in (b) shows anaphase nuclei. (a′) and (a′′) indicate the magnified SAS-4 or γ-tubulin signal of a, respectively; whereas (b′) and (b′′) correspond to the magnified SAS-4 or γ-tubulin signal of b, respectively. Asterisks, intestinal nuclei; arrowheads, SAS-4 or γ-tubulin foci. Scale bar 5 µm. (**B**) and (**C**) L2 wild type animal was stained with DAPI (blue), anti-SAS-4 (green) and anti-α-tubulin (red). (B) shows the intestinal nuclei; whereas lateral hypodermal cells of the same animal in (C). The insets show the α-tubulin signal alone in the framed region in the corresponding panels. Asterisks, intestinal nuclei. Scale bar, 5 µm.
(TIF)

Figure S4 **CDK-2 affects centriole duplication during L1.** (**A**) The intestinal-specific RNAi sensitized animals were subjected to a control RNAi or (**B**) *cdk-2*(*RNAi*) and stained with DAPI (red) or anti-SPD-2 (green). Asterisks indicate the intestinal nuclei and arrowheads point to SPD-2 foci. The insets show high magnification of the regions within the white rectangles. Scale bar, 5 µm. (**C**) The frequency of centriole duplication failure is represented by quantifying the number of intestinal cells that possess two SPD-2 foci 10 hours into the L1 stage. Error bar, standard deviation; n≥50; P<0.05 (t-test). (**D** and **E**) Mass spectrometric analysis of GST::SPD-2 incubated with human CDK2/cyclin A or CDK1/cyclin B, respectively. +80 indicates the phosphorylated amino acid and the arrow highlights S545 in red.
(TIF)

Figure S5 **PLK-1 affects the intestinal nuclear division, but not centriole duplication during the L1 stage.** (**A**) Mass spectrometric analysis of GST::SPD-2 incubated with human PLK-1. +80 indicates the phosphorylated amino acid and the arrow indicates the position of S357 in red. (**B**) The intestinal-specific RNAi sensitized animals were subjected to control RNAi or (**C**) *plk-1*(*RNAi*) and stained with DAPI (red) or anti-SPD-2 (green). Asterisks indicate the intestinal nuclei and arrowheads point to SPD-2 foci. The insets show high magnification of the regions within the white rectangles. Scale bar, 5 µm. (**D**) The frequency of nuclear division failure is represented by quantifying undivided nuclei with two SPD-2 foci. Error bar, standard deviation; n≥50; P<0.05 (t-test).
(TIF)

Form S1 **The primers used for quantifying the relative mRNA abundance of the genes essential for centriole duplication or centrosomal function.** Two pairs of primers were designed for each tested gene except for elt-2 and htp-3 (one pair). The primer sequences were designed and analyzed with the on-line tool available at www.basic.northwestern.edu/biotools/oligocalc.html.
(XLS)
