# Supplementary figures and images for: Centrosome/Cell Cycle Uncoupling and Elimination in the Endoreduplicating Intestinal Cells of C. elegans
Source: PLoS One. 2014 Oct 31;9(10):e110958. doi: 10.1371/journal.pone.0110958 (PMC4215990; doi:10.1371/journal.pone.0110958)

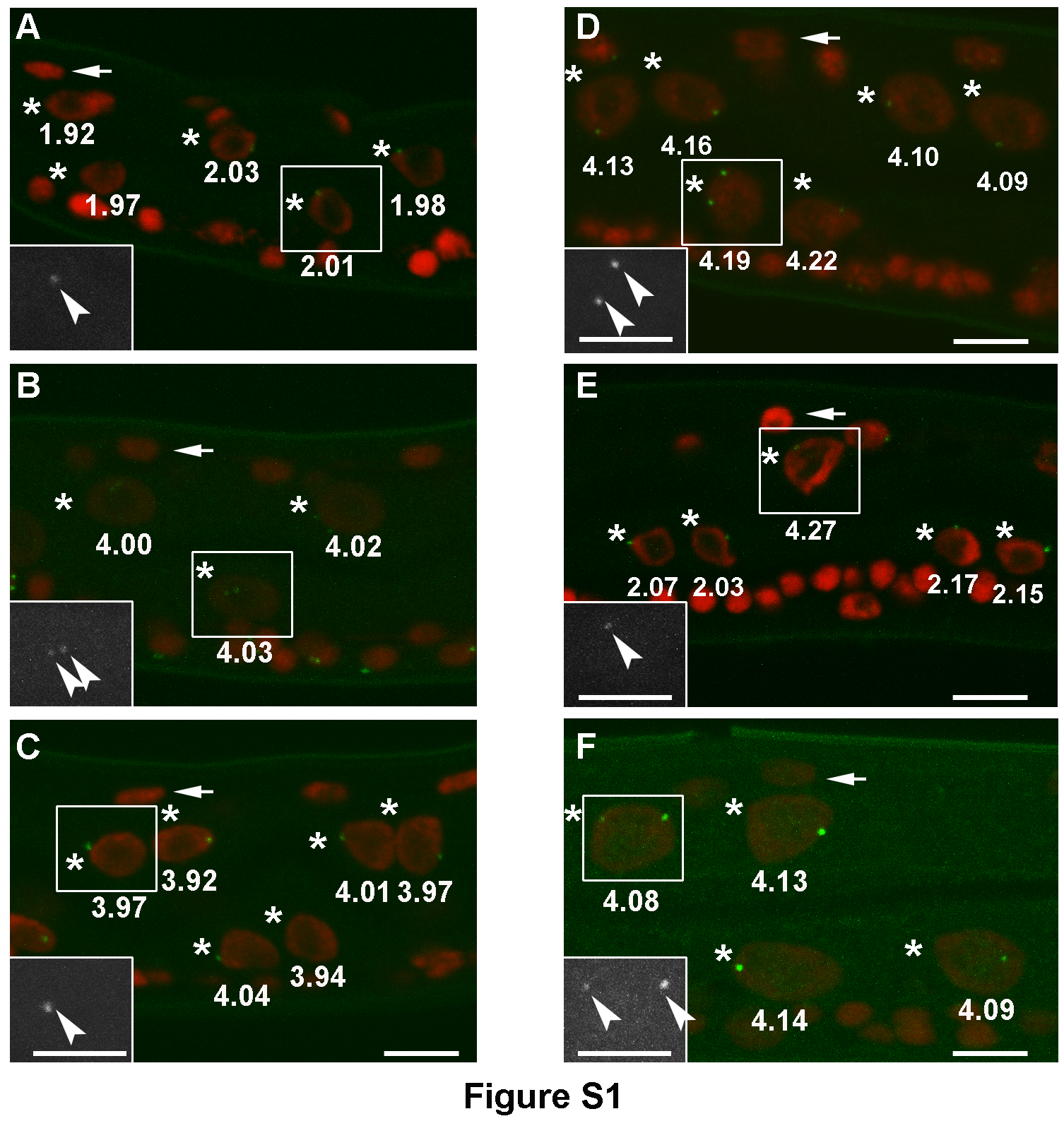

Supplement: Figure S1 — The coupling of centriole duplication with S-phase is regulated developmentally and can be genetically altered. (A–C) spd-2 (oj29) animals carrying the wild type SPD-2::GFP were stained with PI (Red) in the L1 and L2. (D) lin-35 (n745) animals were stained with PI (red) and anti-SPD-2 (green) in the L2. (E) spd-2 (oj29) animals carrying the SPD-2S545A variant were stained with PI (Red) at the L1/L2 transition. (F) spd-2 (oj29) animals carrying the SPD-2S545E variant were stained with PI (Red) in mid-L2 stage. The asterisks indicate the intestinal nuclei and the arrow heads point out the SPD-2 foci. The arrows indicate the muscle cells that are used as the 2C reference and the numbers under the intestinal nuclei indicates their DNA content. Scale bar, 5 µm. (TIF) [file pone.0110958.s001.tif]

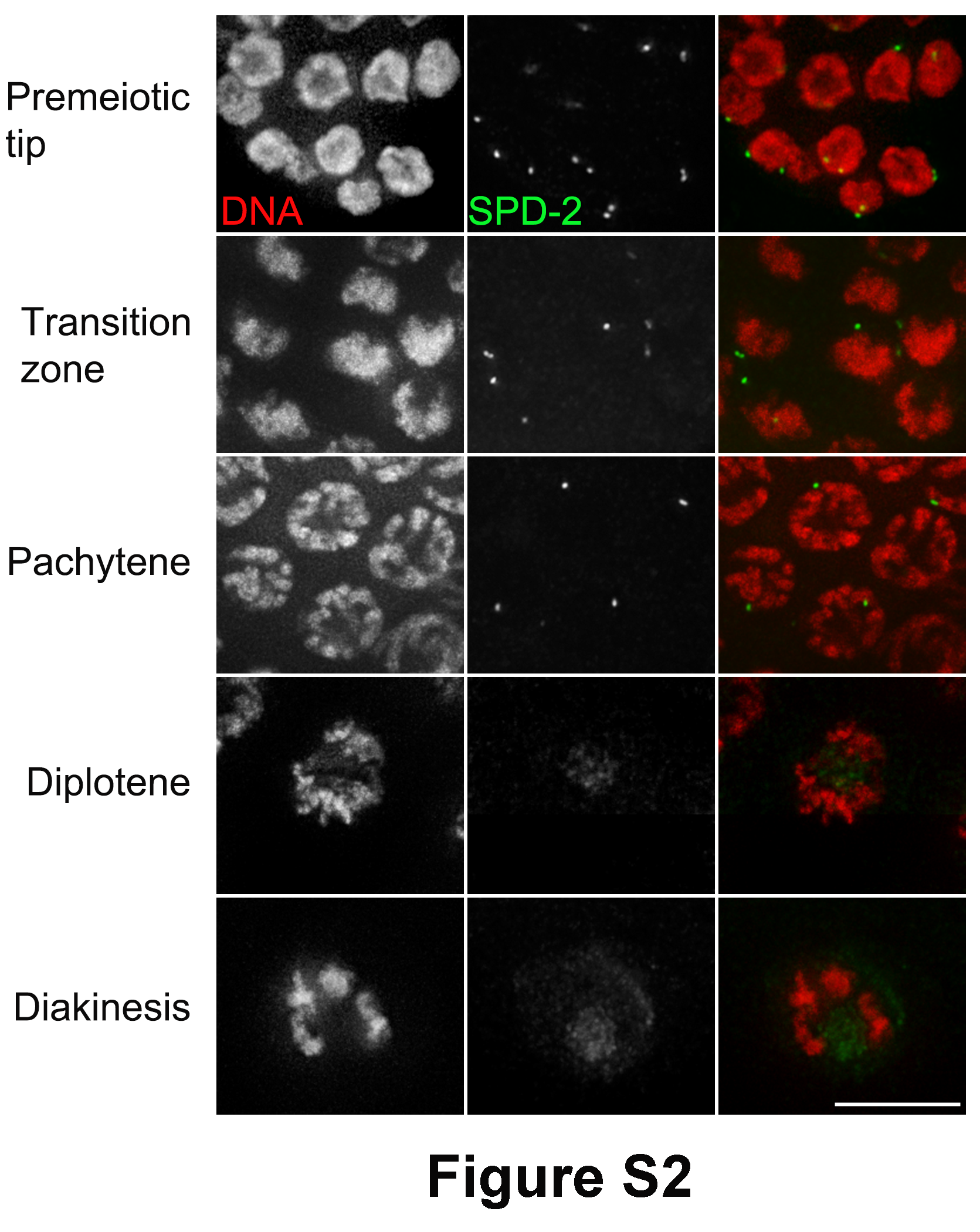

Supplement: Figure S2 — SPD-2 diffuses into nuclei before its elimination in the germ line. The gonad of a N2 young adult was dissected and stained with DAPI (red) and SPD-2 (green). Scale bar, 5 µm. (TIF) [file pone.0110958.s002.tif]

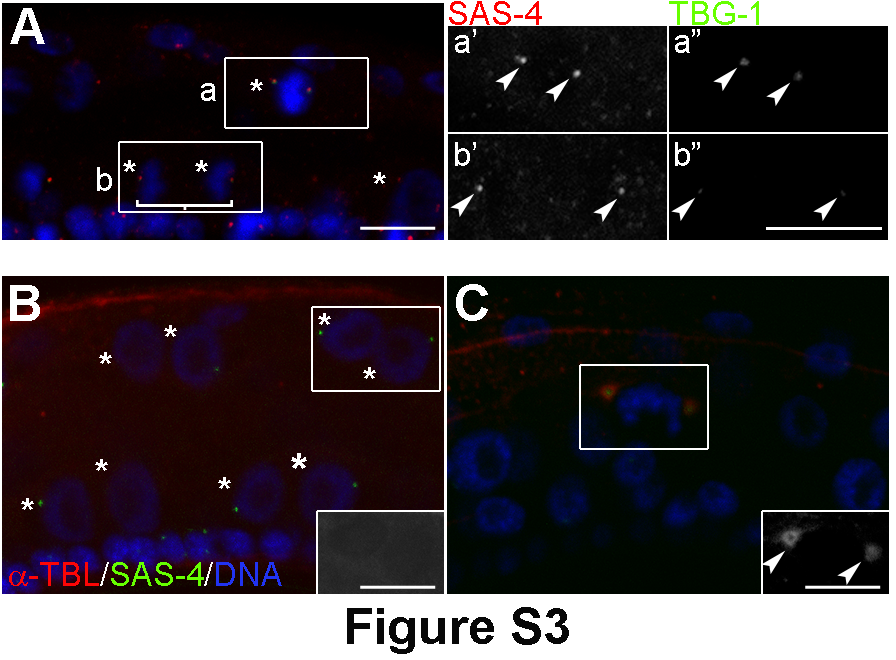

Supplement: Figure S3 — γ-tubulin dispersal from the centriole affects its MTOC function. (A) N2 at late L2 were stained with DAPI (blue), anti-SAS-4 (red) and anti-γ-tubulin (green). (a) highlights a metaphase nucleus while the lineage bracket in (b) shows anaphase nuclei. (a′) and (a′′) indicate the magnified SAS-4 or γ-tubulin signal of a, respectively; whereas (b′) and (b′′) correspond to the magnified SAS-4 or γ-tubulin signal of b, respectively. Asterisks, intestinal nuclei; arrowheads, SAS-4 or γ-tubulin foci. Scale bar 5 µm. (B) and (C) L2 wild type animal was stained with DAPI (blue), anti-SAS-4 (green) and anti-α-tubulin (red). (B) shows the intestinal nuclei; whereas lateral hypodermal cells of the same animal in (C). The insets show the α-tubulin signal alone in the framed region in the corresponding panels. Asterisks, intestinal nuclei. Scale bar, 5 µm. (TIF) [file pone.0110958.s003.tif]

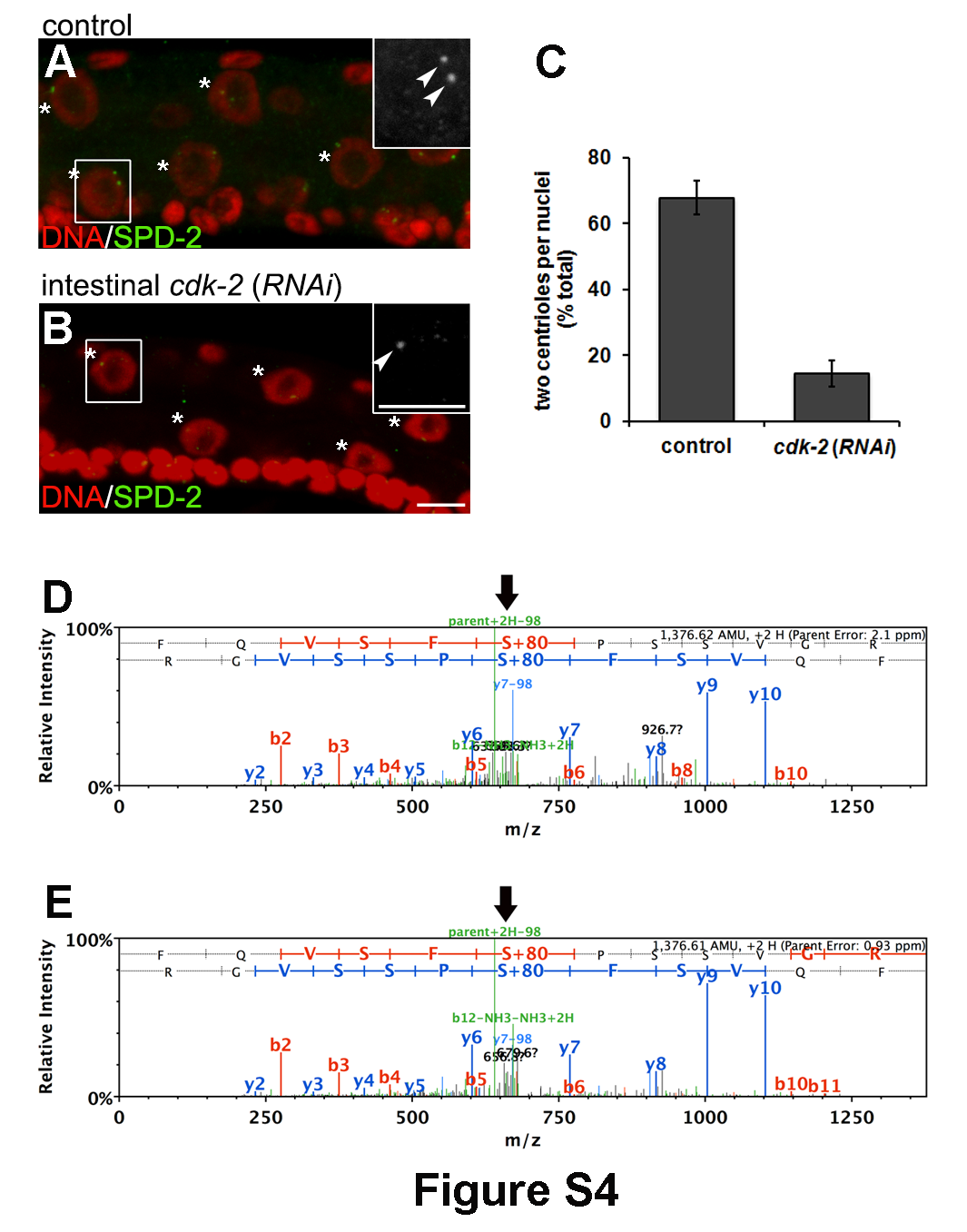

Supplement: Figure S4 — CDK-2 affects centriole duplication during L1. (A) The intestinal-specific RNAi sensitized animals were subjected to a control RNAi or (B) cdk-2(RNAi) and stained with DAPI (red) or anti-SPD-2 (green). Asterisks indicate the intestinal nuclei and arrowheads point to SPD-2 foci. The insets show high magnification of the regions within the white rectangles. Scale bar, 5 µm. (C) The frequency of centriole duplication failure is represented by quantifying the number of intestinal cells that possess two SPD-2 foci 10 hours into the L1 stage. Error bar, standard deviation; n≥50; P<0.05 (t-test). (D and E) Mass spectrometric analysis of GST::SPD-2 incubated with human CDK2/cyclin A or CDK1/cyclin B, respectively. +80 indicates the phosphorylated amino acid and the arrow highlights S545 in red. (TIF) [file pone.0110958.s004.tif]

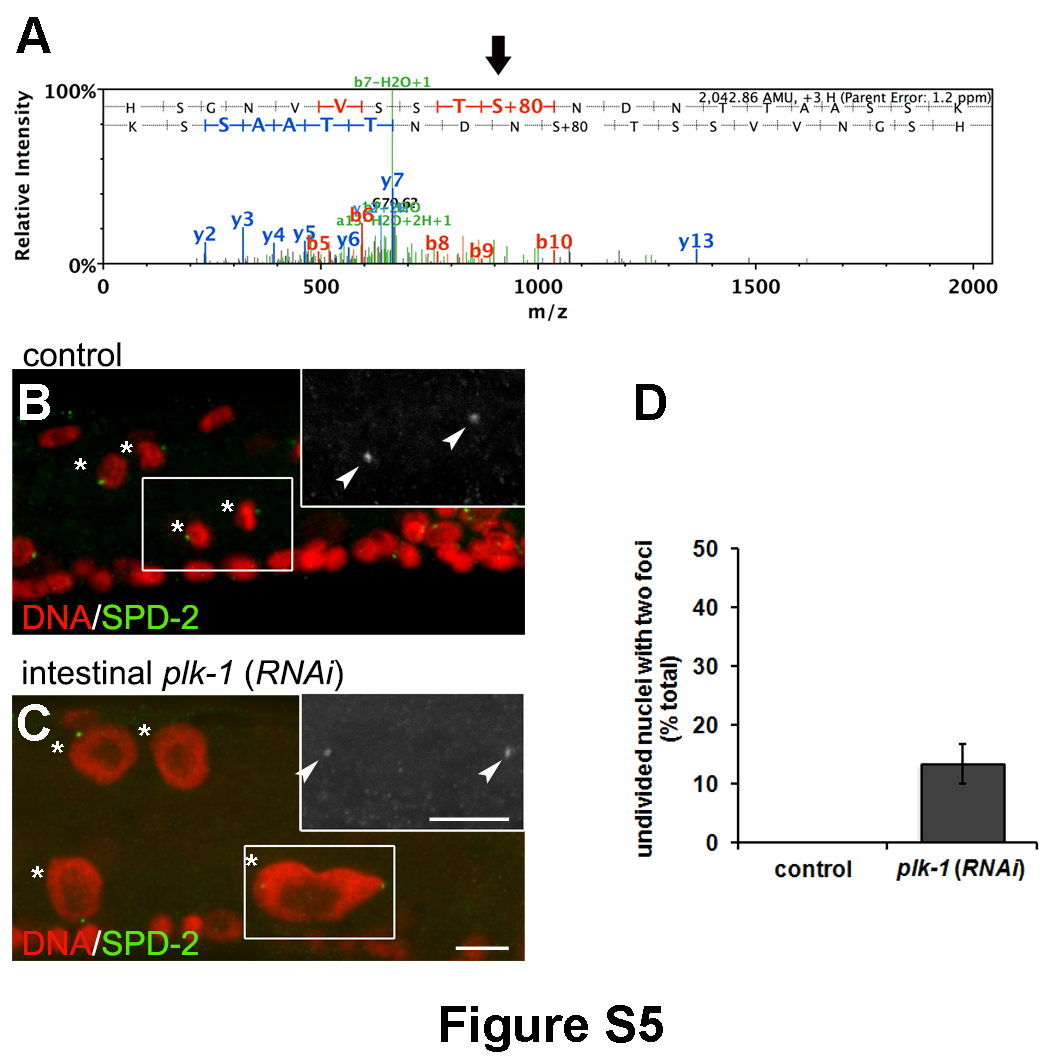

Supplement: Figure S5 — PLK-1 affects the intestinal nuclear division, but not centriole duplication during the L1 stage. (A) Mass spectrometric analysis of GST::SPD-2 incubated with human PLK-1. +80 indicates the phosphorylated amino acid and the arrow indicates the position of S357 in red. (B) The intestinal-specific RNAi sensitized animals were subjected to control RNAi or (C) plk-1(RNAi) and stained with DAPI (red) or anti-SPD-2 (green). Asterisks indicate the intestinal nuclei and arrowheads point to SPD-2 foci. The insets show high magnification of the regions within the white rectangles. Scale bar, 5 µm. (D) The frequency of nuclear division failure is represented by quantifying undivided nuclei with two SPD-2 foci. Error bar, standard deviation; n≥50; P<0.05 (t-test). (TIF) [file pone.0110958.s005.tif]
